# Spatial and Epigenetic Polarity of the AR Promoter During Prostate Cancer Progression: An In Silico Analysis

**DOI:** 10.3390/ijms27198822

**Published:** 2026-10-02

**Authors:** Aleksey M. Krasnyi, Roman A. Ivanov, Ekaterina Minskaia

**Affiliations:** Translational Medicine Research Center, Sirius University of Science and Technology, Federal Territory Sirius, Olympic Ave. 1, 354340 Sirius, Russia; ivanov.ra@talantiuspeh.ru (R.A.I.); minskaya.es@talantiuspeh.ru (E.M.)

**Keywords:** androgen receptor, prostate cancer, AR promoter, DNA methylation, FOXA1, H3K27ac, lineage plasticity, PRC1.1, H3K27me3, transcription factors

## Abstract

The androgen receptor (AR) is a key regulator of prostate cancer progression, yet the relationship between AR promoter methylation, chromatin state, and receptor expression across distinct tumor phenotypes remains incompletely understood. We performed an integrative in silico analysis of the AR promoter region spanning −2000 to +500 bp relative to the transcription start site (TSS), combining motif predictions for 24 transcription factors (TFs) with DNA methylation, TF ChIP-seq, and histone modification profiles from normal prostate tissue, primary and metastatic tumors, and cell models. Integration of these datasets revealed pronounced spatial heterogeneity in the epigenetic organization of the AR promoter. The distal segment contained predicted motifs for the selected lineage-associated TFs FOXA1, GATA2, HOXB13, and NKX3-1 and showed substantial variability in DNA methylation. Reduced methylation at selected distal sites or regions was observed in primary adenocarcinoma and in AR-low and AR-negative tumors and cell models, including tumors with a neuroendocrine phenotype. In TCGA-PRAD primary tumors, between-sample differences in AR promoter methylation explained only a small proportion of the variation in AR mRNA levels. Within the distal FOXA1 motif cluster (−751 to −678 bp), within-sample-normalized FOXA1 signal was higher in primary tumors than in matched normal tissue and was further elevated in patient-derived xenografts (PDXs) originating from metastatic castration-resistant prostate cancer (mCRPC). Exploratory analysis of matched normal–tumor pairs revealed a positive correlation between changes in distal FOXA1 signal and changes in proximal H3K27ac signal. AR-positive C4-2B cells retained proximal H3K27ac without detectable FOXA1 occupancy at the distal cluster, consistent with an alternative active promoter configuration. The proximal TSS-associated segment was generally hypomethylated but displayed heterogeneous chromatin states. The −280 to +100 bp region was a recurrent focus of H3K27ac enrichment. In VCaP cells, proximal AR and ERG ChIP-seq signals overlapped, although no AR motif meeting the screening threshold was detected in this region. H3K27me3 repressive mark profiles showed marked heterogeneity across the examined models and tissues. Among models used as normal controls, RWPE-1 and PrEC showed H3K27me3 enrichment around the AR TSS, whereas primary normal prostate tissue showed no comparable enrichment. Heterogeneity was also evident between AR-negative cell lines: PC-3 showed pronounced proximal H3K27me3 enrichment, whereas DU145 lacked comparable enrichment. The analyzed mCRPC metastases likewise did not exhibit a profile comparable to that of PC-3. In AR-negative PC-3 cells, the proximal region additionally showed enrichment of KDM2B, PCGF1, and RNF2, together with strong H2AK119ub1 enrichment. These findings support a model in which the AR promoter comprises an epigenetically plastic distal segment and a proximal TSS-associated regulatory region characterized by context-dependent TF occupancy and chromatin states. This heterogeneity, together with the limited explanatory value of promoter methylation for AR mRNA levels, is consistent with DNA methylation alone being insufficient to account for differences in AR expression across prostate cancer phenotypes.

## 1. Introduction

Androgen receptor (AR) signaling plays a central role in maintaining the luminal identity of prostate epithelial cells and in the development and progression of prostate cancer (PCa) [1,2,3]. Androgen deprivation therapy and contemporary agents targeting the AR signaling pathway, including the AR antagonist enzalutamide and the androgen biosynthesis inhibitor abiraterone, initially provide substantial antitumor activity [4]. However, patients with advanced PCa eventually develop castration-resistant prostate cancer (CRPC), including its metastatic form (mCRPC), in which AR activity is frequently maintained or restored through amplification of the AR locus, increased receptor expression, acquisition of activating mutations, and generation of constitutively active splice variants [2,4].

Under sustained therapeutic pressure, a subset of tumors acquires the ability to alter cellular identity and transition toward states with reduced or complete loss of AR dependence [5,6]. Such changes in cell state represent manifestations of lineage plasticity and are accompanied by extensive transcriptional reprogramming [5,7]. Studies of metastatic CRPC have also revealed pronounced differences in the epigenomic landscape, including DNA methylation changes, among tumors with distinct AR and neuroendocrine signaling states [8,9]. Extreme manifestations of lineage plasticity include neuroendocrine prostate cancer (NEPC), including the small-cell phenotype, and double-negative prostate cancer (DNPC; AR−/NE−) [5,8]. Experimental studies have demonstrated that combined loss of TP53 and RB1 promotes activation of a SOX2-dependent lineage-plasticity program and resistance to antiandrogen therapy [6]. Epigenetic reprogramming, including alterations in DNA methylation, represents one potential mechanism linking therapeutic pressure to changes in tumor transcriptional state.

Historically, regulation of AR expression has been considered largely within a binary model in which hypermethylation of the promoter CpG island results in transcriptional silencing. Such an association was demonstrated in several AR-negative prostate cancer cell lines, including DU145, DuPro, TSU-PR1, and PPC-1, in which methylation of the promoter-associated CpG island was accompanied by loss of AR expression [10]. Subsequent bisulfite mapping identified two preferentially methylated regions close to the transcription start site, spanning −131 to −121 bp and +44 to +54 bp; methylation of these regions was also associated with loss of AR expression [11]. However, more recent evidence points to a substantially more complex epigenetic organization associated with prostate cancer progression. Genome-wide analysis of metastatic CRPC revealed extensive heterogeneity in DNA methylation and an association between increased AR expression and hypomethylation of several regions within the AR locus, including a promoter-adjacent region, a previously characterized distal enhancer, and additional regulatory elements located upstream and downstream of the gene [9]. In a study of bone metastases by Ylitalo et al. using the Infinium MethylationEPIC array, promoter-associated CpG sites showed an overall reduction in methylation in metastatic lesions compared with primary tumors. This trend involved individual CpG sites within the AR locus and was accompanied by increased levels of full-length AR transcripts [12].

The AR promoter lacks canonical TATA and CAAT boxes, is GC-rich, and contains several closely spaced transcription initiation sites [13,14]. Basal transcriptional activity of the proximal promoter depends, in part, on GC-rich regulatory elements and binding of the TF SP1 [13,14]. In addition to DNA methylation, promoter activity is influenced by chromatin accessibility, histone modifications, and the binding of transcriptional and architectural proteins. The latter include CTCF, which contributes to three-dimensional genome organization, chromatin interactions, and transcriptional regulation [15]. In specific genomic contexts, CTCF binding may also influence RNA polymerase II (RNA Pol II) pausing and co-transcriptional splicing [16]. Nevertheless, the spatial organization of regulatory elements within the AR promoter and its remodeling during prostate cancer progression remain insufficiently understood.

The aim of the present study was to map potential TF-binding sites within an extended AR promoter region spanning −2000 to +500 bp relative to the transcription start site, to characterize its spatial organization, and to determine how local regulatory features of the promoter are associated with distinct AR signaling states and transition toward an AR-negative neuroendocrine phenotype. To this end, motif analysis was integrated with publicly available data on DNA methylation, TF binding, and histone modifications.

## 2. Results

### 2.1. Literature-Based Selection of Candidate Transcription Factors

The AR gene encodes the androgen receptor, a ligand-activated nuclear TF whose expression level and activity substantially influence the sensitivity of prostate cancer cells to androgen-deprivation therapy and contribute to the development of castration-resistant prostate cancer (CRPC) [1,2,4]. The AR promoter lacks a canonical TATA box and is characterized by a high GC content [13,14]. Against this background, AR transcription may be regulated by a broad spectrum of TFs, including components of the basal transcriptional machinery as well as lineage-specific, proliferative, stress-responsive, and pro-inflammatory regulators.

To provide a biological rationale for the candidate TF panel used in the subsequent promoter analysis, we considered published experimental evidence linking individual factors to AR transcription or AR-locus regulation. The principal experimental evidence and references are summarized in Table 1.

#### 2.1.1. Basal Transcriptional Regulation of the AR Promoter: The Role of SP1

The proximal promoter of the AR gene is TATA-independent and contains a GC box directly bound by the TF SP1 [13,14]. Functional analyses have shown that disruption of SP1 binding alters the selection of transcription initiation sites, supporting its role in the basal regulation of AR expression [13].

#### 2.1.2. Pioneer and Lineage-Specific Factors Regulating the AR Locus

FOXA1 is a pioneer TF associated with the maintenance of luminal differentiation and the AR-dependent transcriptional program. In clinical prostate cancer specimens, FOXA1 and AR protein levels were positively correlated [17]. However, the functional effect of FOXA1 on expression of the AR gene itself appears to be context-dependent. FOXA1 depletion in hormone-deprived LNCaP cells increased AR mRNA levels and moderately increased AR protein expression [18], whereas in 22Rv1 cells it increased the expression of specific AR variants, including AR-V7, without substantially increasing the full-length AR transcript [19].

GATA2 is a pioneer factor that positively regulates AR expression. GATA2 binding has been identified at several regulatory regions within the AR locus, including upstream and promoter-proximal regions. GATA2 depletion reduced AR mRNA and protein levels both before and after androgen stimulation, indicating a role for GATA2 in maintaining basal and hormone-induced AR expression [20].

HOXB13 is a prostate lineage-defining TF functionally linked to AR expression. HOXB13 was identified in a functional CRISPR-Cas9 screen as a regulator of AR transcription, and subsequent suppression of HOXB13 reduced AR mRNA and protein levels in AR-positive prostate cancer models [21].

TFAP2C was likewise identified in the functional CRISPR-Cas9 screen as a factor required for maintaining AR expression. Suppression of TFAP2C reduced AR mRNA and protein levels in AR-positive cell models [21].

Expression of the prostate-specific homeobox factor NKX3-1 correlated with AR expression in normal and malignant prostate tissue [22]. Functional experiments demonstrated that NKX3-1 and AR form a positive reciprocal regulatory loop: NKX3-1 binds an intragenic region of AR located approximately 79 kb downstream of the TSS, and its depletion with two independent siRNAs reduces both AR mRNA and protein levels [23].

#### 2.1.3. Autoregulation of AR Expression and GR-Mediated Bypass of Antiandrogen Blockade

Ligand-activated AR can participate in the negative regulation of its own expression. A non-consensus androgen-responsive element has been identified within the 5′ untranslated region (UTR) of the AR gene, and AR binding to this element was confirmed by ChIP analysis. Mutation of this element attenuated androgen-dependent repression of transcriptional activity, supporting its functional role in negative autoregulation of AR [24]. A second AR-binding regulatory element has been identified in intron 2 of the AR gene. At high androgen levels, AR binding to this region was accompanied by recruitment of LSD1, demethylation of H3K4me1/2, and repression of AR transcription [25].

The glucocorticoid receptor NR3C1 (GR) exhibits DNA-binding specificity that overlaps with that of AR and can regulate a subset of common target genes. In models of enzalutamide resistance, increased GR expression enabled functional substitution for AR: activated GR maintained the expression of a subset of AR-regulated genes and contributed to the resistant phenotype. GR activation by dexamethasone promoted enzalutamide resistance, whereas genetic or pharmacological GR inhibition restored sensitivity to antiandrogen therapy [26]. However, direct GR binding to the AR promoter and a direct effect of GR on AR expression have not been experimentally established.

#### 2.1.4. CTCF and Spatial Organization of the AR Locus

CTCF is an architectural protein involved in the spatial organization of chromatin and interactions between distant regulatory regions. Comparative in situ Hi-C analysis of normal prostate epithelial RWPE-1 cells and CRPC 22Rv1 cells revealed cancer-specific enhancer–promoter interactions within the AR locus, together with the formation of additional smaller chromatin domains. CTCF-binding sites were located at the boundaries of these domains; however, CTCF occupancy profiles did not differ substantially between RWPE-1 and 22Rv1 cells [27].

Analysis of RNA Pol II-associated chromatin interactions identified an active enhancer cluster approximately 700 kb upstream of the AR promoter that interacts with the promoter in LNCaP and VCaP cells. Amplification of the AR locus in VCaP cells was associated with an increased number of regulatory interactions and increased AR transcription. Enhanced CTCF binding was also observed in the 3′ region of the AR gene in these cells. It was proposed that the formation of new insulating elements within the amplified locus could restrict transcription from individual gene copies and contribute to the generation of truncated AR variants; however, this hypothesis has not been functionally tested [28].

#### 2.1.5. Proliferation- and Cell Plasticity-Associated Factors in the Regulation of AR Expression

E2F1 participates in the negative regulation of AR transcription. Increased E2F1 expression in prostate epithelial cells reduced AR mRNA and protein levels as well as the activity of an AR promoter reporter. ChIP analysis detected E2F1 and the pocket proteins p107 and p130 at the AR promoter [29]. A subsequent study demonstrated that repression of AR transcription may involve the E2F1–DNMT1 axis: DNMT1 was associated with the minimal AR promoter, while repression occurred without detectable changes in promoter methylation [30]. Thus, E2F1 likely participates in a repressive complex at the AR promoter rather than necessarily acting through a canonical E2F-binding motif.

MYC is a positive regulator of full-length AR and AR splice-variant expression. MYC depletion in 22Rv1, LNCaP95, and VCaP cells reduced AR pre-mRNA, mature AR-FL and AR-V transcripts, and the activity of reporter constructs containing regulatory regions of the AR gene [31].

In LNCaP-AR cells, ChIP-qPCR revealed DHT-dependent binding of KLF4 to sites located at −347 and +647 bp relative to the TSS. KLF4 depletion reduced AR mRNA and protein levels in LNCaP and 22Rv1 cells [32].

KLF5 also directly promotes AR transcription. KLF5 depletion reduced AR mRNA and protein levels in LNCaP and C4-2B cells and decreased the activity of a reporter construct containing the −2000 to +200 bp region of the AR promoter. ChIP analysis confirmed KLF5 binding to the proximal promoter region containing sites between −107 and −93 bp [33].

#### 2.1.6. ERG and Other ETS Factors in the Regulation of AR Expression

ERG has been identified as a negative regulator of AR expression. In prostate cancer, increased ERG expression is frequently associated with the TMPRSS2–ERG chromosomal rearrangement. In VCaP cells, ERG binding to the AR promoter region was identified by ChIP-seq and confirmed by ChIP-PCR. ERG overexpression reduced AR mRNA and protein levels in VCaP, LNCaP, and 22Rv1 cells, whereas ERG depletion in VCaP cells increased AR expression. ERG also reduced the activity of an AR promoter reporter, supporting a direct repressive effect on AR transcription [34].

Functional evidence for ETS1 is more limited. In a study of the miR-204 regulatory network, ETS1 depletion reduced AR mRNA levels in LNCaP and VCaP cells and was accompanied by increased methylation of the AR promoter CpG region. Direct binding of ETS1 to the AR promoter was not examined [35].

#### 2.1.7. c-Jun/AP-1 in the Negative Regulation of AR Expression

c-Jun has been shown to suppress AR expression. c-Jun overexpression reduced the activity of a reporter construct containing the AR promoter [36]. In LNCaP cells, inducible c-Jun expression decreased both mature and nascent AR RNA levels and, following prolonged expression, also reduced AR protein levels [37]. These findings support transcriptional repression of AR by c-Jun, although direct c-Jun occupancy of the AR promoter was not demonstrated in these studies.

#### 2.1.8. STAT Signaling in the Regulation of AR Expression and Activity

STAT1 signaling has been associated with progression toward AR-low/AR-independent, therapy-resistant prostate cancer states. Increased STAT1 expression is inversely associated with AR levels and accompanies the acquisition of stem-like and neuroendocrine features in several prostate cancer models, including TP53/RB1-deficient cells [38].

Among STAT family members, the strongest functional evidence for regulation of AR transcription has been obtained for STAT5. STAT5 suppression reduced newly synthesized AR pre-mRNA as well as full-length AR and AR splice variants in several prostate cancer models, whereas constitutively active STAT5 increased AR transcription [39].

#### 2.1.9. NF-κB in Context-Dependent Regulation of AR Expression

The canonical NF-κB complex formed by RELA/p65 and NFKB1/p50 has been shown to participate in the regulation of AR transcription. In LNCaP cells, overexpression of p65, as well as combined expression of p65 and p50, increased the activity of an AR promoter reporter in a dose-dependent manner. Increased p65 expression was accompanied by higher AR mRNA and protein levels, whereas inhibition of NF-κB reduced AR expression. ChIP-PCR detected binding of p50-containing complexes to several regions within the 5′ regulatory region of AR [40].

NF-κB2/p52 has been shown to promote ligand-independent AR signaling and the expression of AR splice variants, including AR-V7, without a corresponding substantial increase in full-length AR. NFKB2/p52 should therefore be considered primarily a regulator of AR activity and AR splice-variant generation [41].

#### 2.1.10. Hypoxia-Response Factors and TP53-Dependent Regulation of AR

In clinical prostate cancer specimens, AR expression was positively associated with the hypoxia-response factors HIF-1α and HIF-2α. Immunohistochemical analysis of a tissue microarray comprising 149 radical prostatectomy specimens demonstrated an association between HIF-1α and AR expression at *p* = 0.04, whereas the association between HIF-2α and AR was stronger (*p* < 0.001). HIF-1α expression was also correlated with HIF-2α expression [42].

p53 is a negative regulator of AR expression. Activation of p53 in LNCaP cells and normal prostate epithelial cells reduced AR mRNA and protein levels. Forced p53 expression also suppressed AR expression, whereas p53 depletion resulted in increased AR levels. Following genotoxic activation of p53 with etoposide, ChIP-PCR detected p53 association with the 5′ regulatory region of the *AR* gene containing a putative p53-responsive element approximately −488 to −469 bp relative to the TSS. No such binding was detected in untreated cells. These findings support stress-induced negative regulation of *AR* transcription by p53 but do not indicate constitutive occupancy of the promoter under basal conditions [43].

### 2.2. Screening of Potential TF-Binding Sites in the AR Promoter Using JASPAR Matrices

#### 2.2.1. Screening Methodology and Overall Landscape of Predicted Motifs

To systematically assess potential TF-binding sites within the AR promoter region, an in silico scan was performed across a 2501-bp sequence spanning −2000 to +500 bp relative to the transcription start site (TSS). In the GRCh38/hg38 coordinate system, the analyzed fragment corresponds to chrX:67,542,021–67,544,521. The selected interval encompasses the distal upstream regulatory region, the proximal TSS-associated promoter segment, and a short region downstream of the TSS.

Two significance tiers were used to retain the most reliable predictions: a strict tier, *p* < 10^−4^, and a supportive tier, 10^−4^ ≤ *p* < 5 × 10^−4^. At the primary selection threshold of *p* < 5 × 10^−4^, 104 motif clusters were identified, including 38 strict and 66 supportive clusters. Overlapping matches for the same TF and DNA strand were represented by a single cluster, as described in Section 4.2. The genomic coordinates, positions relative to the AR TSS, strand, JASPAR matrix identifiers, and nominal *p*-values of all 104 retained motif clusters are provided in Supplementary Table S7. Cluster boundaries and representative motif-site coordinates are reported separately.

For spatial analysis, the promoter was operationally divided at −280 bp relative to the TSS into a distal segment (−2000 to −281 bp) and a proximal segment (−280 to +500 bp). Under this classification, 53 of 104 clusters (51.0%) were located in the distal segment and 51 of 104 clusters (49.0%) in the proximal segment. The TF-level motif-screening results are summarized in Table 2.

As an additional sensitivity analysis, motif-cluster abundance was evaluated against sequence-composition controls. The overall number of JASPAR motif clusters in the AR promoter was not increased relative to GC-matched human promoters, and no individual TF or JASPAR matrix remained significantly enriched after correction for multiple testing. Full details of the background selection, sequence-composition controls, and per-TF/per-matrix results are provided in Supplementary Methods and Supplementary Tables S2–S6.

#### 2.2.2. Spatial Polarization of Predicted Regulatory Motifs

The distribution of predicted motifs differed across the AR promoter in both density and TF composition. Using the operational −280 bp boundary, the distal segment (−2000 to −281 bp; 1720 bp) contained 53 of 104 primary motif clusters (51.0%), whereas the proximal segment (−280 to +500 bp; 781 bp) contained 51 clusters (49.0%). Because the two segments differ substantially in length, these values correspond to motif-cluster densities of 30.8 and 65.3 clusters/kb, respectively.

The proximal segment contained a high proportion of motif matches for SP1, KLF4, and KLF5. Of the 51 proximal clusters, 29 corresponded to these three factors: 11 clusters for SP1, 10 for KLF4, and 8 for KLF5, together accounting for 56.9% of the proximal predictions. The proximal segment additionally contained six TFAP2C clusters, six ERG clusters, three CTCF clusters, two clusters each for E2F1, ETS1, and RELA, and one NFKB2 cluster.

In contrast, predicted motifs for the lineage-associated factors FOXA1, GATA2, HOXB13, and NKX3-1 were confined to the distal segment. Motifs for the nuclear receptors AR and NR3C1 and for JUN, STAT1, STAT5, and TP53 were also located exclusively in this segment. The largest functional groups in the distal segment comprised STAT1/STAT5 (13 clusters), FOXA1/GATA2/HOXB13/NKX3-1 (12 clusters), and AR/NR3C1 (10 clusters).

**Figure 1:**
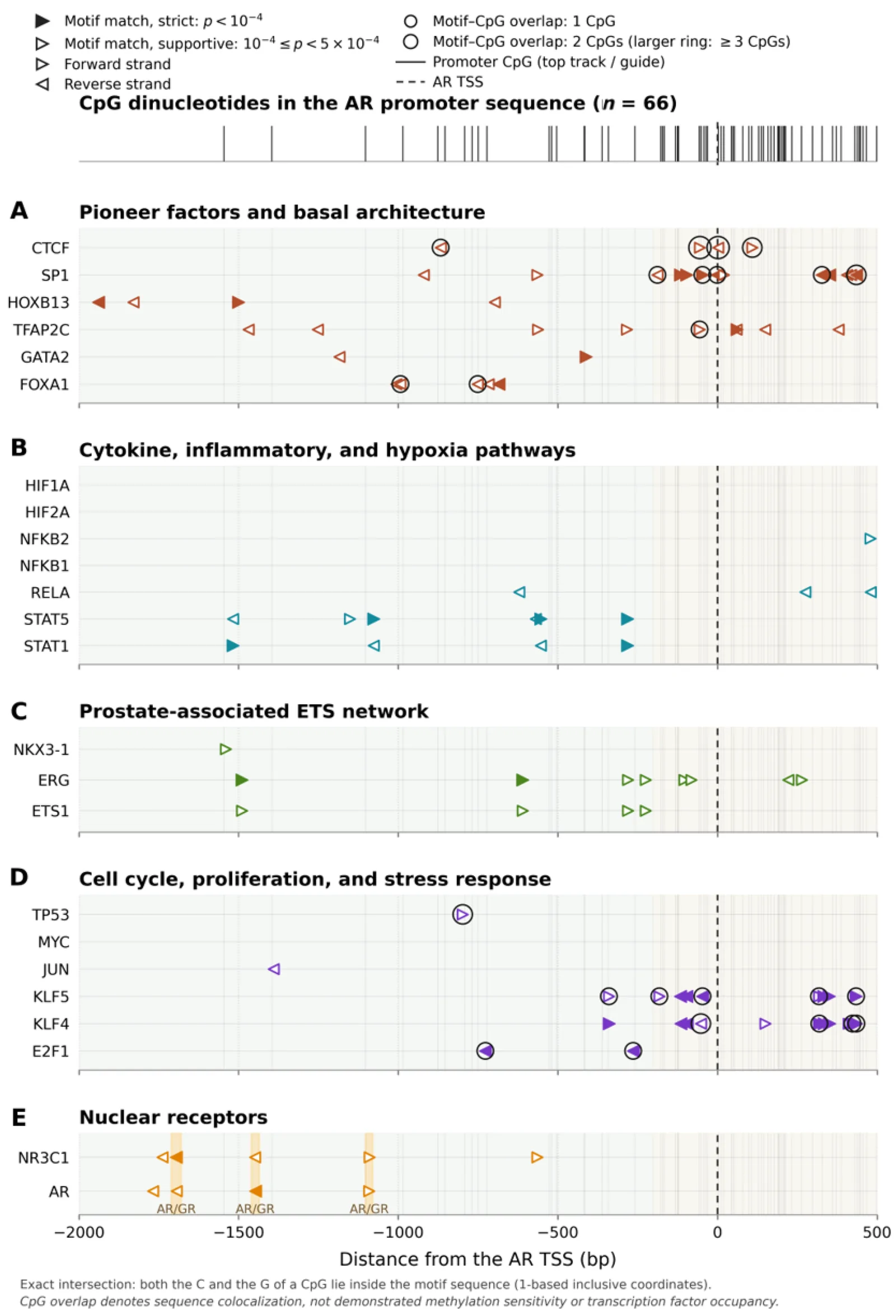
Spatial distribution of predicted motifs for 24 TFs and CpG dinucleotides across the extended *AR* promoter region. Panels show (**A**) pioneer factors and basal architecture; (**B**) cytokine, inflammatory, and hypoxia pathways; (**C**) the prostate-associated ETS network; (**D**) cell-cycle, proliferation, and stress response; and (**E**) nuclear receptors. Triangles indicate motif matches at *p* < 5 × 10^−4^. Filled symbols denote strict matches (*p* < 10^−4^), whereas open symbols denote supportive matches (10^−4^ ≤ *p* < 5 × 10^−4^). Triangle orientation indicates the forward or reverse strand. Rings mark motif intervals containing one or more CpG dinucleotides; ring size reflects the number of CpGs within the motif interval. Vertical lines indicate the positions of the 66 CpG dinucleotides in the analyzed sequence. The dashed vertical line at coordinate 0 indicates the *AR* TSS. Abbreviations: CpG, cytosine–phosphate–guanine dinucleotide; TF, transcription factor; TSS, transcription start site; bp, base pairs.

Several factors showed mixed spatial distributions. TFAP2C had four distal and six proximal clusters, CTCF one distal and three proximal clusters, E2F1 two distal and two proximal clusters, and ETS1 two distal and two proximal clusters. ERG was predominantly represented in the proximal segment, with two distal and six proximal clusters, whereas RELA included one distal and two proximal clusters. SP1, KLF4, and KLF5 also showed a marked proximal bias. No motif matches meeting the primary threshold of *p* < 5 × 10^−4^ were detected for MYC, NFKB1, HIF1A, or HIF2A/EPAS1 within the analyzed interval. The spatial relationship between predicted TF-binding motifs and CpG dinucleotides within the analyzed promoter interval is also illustrated in Figure 1.

These sequence-based predictions were used to map candidate TF-binding positions rather than to assess motif enrichment or TF occupancy. Where independent ChIP-seq data were available, predicted regions were subsequently evaluated against experimentally observed promoter occupancy.

### 2.3. Methylation Patterns of the AR Promoter Across Distinct Phenotypic States of Prostate Cancer

To evaluate the relationship between methylation of the *AR* promoter region, tumor progression, and changes in the AR-dependent phenotype, three independent datasets were compared: the primary prostate adenocarcinoma cohort TCGA-PRAD (The Cancer Genome Atlas Prostate Adenocarcinoma), the C4-2B and NCI-H660 cell models from GSE158598, and the clinical metastatic CRPC cohort of the West Coast Dream Team (WCDT). The analysis was performed within the −2000 to +500 bp interval relative to the AR transcription start site (TSS), previously used for mapping potential TF-binding sites.

#### 2.3.1. AR Promoter Methylation in the TCGA-PRAD Cohort

**Figure 2:**
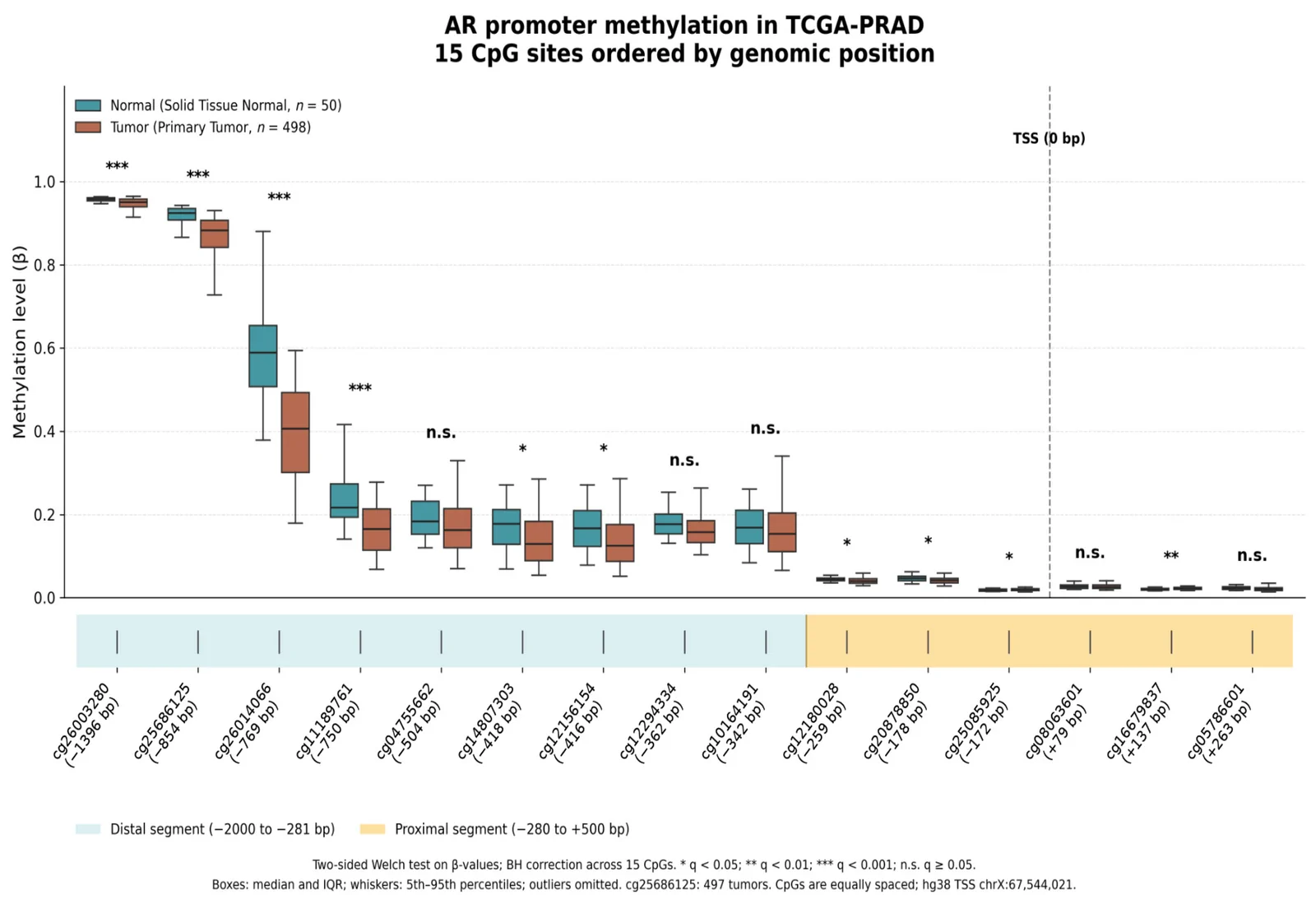
Methylation levels (β-values) of 15 CpG sites within the AR promoter region in TCGA-PRAD samples (Primary Tumor, *n* = 498; Solid Tissue Normal, *n* = 50; cg25686125, *n* = 497 tumors). Boxplots show the median, interquartile range (25–75%), and 5–95% whiskers. CpG sites are ordered by position relative to the AR TSS. Significance symbols in the figure refer to the group-wise Welch test on β-values with Benjamini–Hochberg correction across the 15 CpGs. * *q* < 0.05; ** *q* < 0.01; *** *q* < 0.001; n.s., *q* ≥ 0.05. Exact *q* values are reported in Table 3. The −280 bp coordinate is retained as an operational distal/proximal boundary for cross-dataset integration; probes at −259 bp and downstream are classified as proximal. Abbreviations: BH, Benjamini–Hochberg; CpG, cytosine–phosphate–guanine dinucleotide; IQR, interquartile range; TCGA-PRAD, The Cancer Genome Atlas Prostate Adenocarcinoma; TSS, transcription start site; bp, base pairs.

To assess methylation changes associated with primary tumor transformation, Illumina HumanMethylation450K data from the analyzed TCGA-PRAD matrix were examined for 15 prespecified CpG sites within the AR promoter interval. Methylation data were available for 498 primary tumors and 50 morphologically normal solid prostate tissue samples adjacent to tumor; one tumor value was missing for cg25686125 (*n* = 497). Group-wise tumor-normal differences were summarized as mean β-value differences (Δβ), while the primary paired sensitivity analysis used the 50 patients with both tumor and normal methylation data and tested M-values. These methylation profiles are shown in Figure 2.

In normal prostate tissue, the assayed CpGs showed a graded spatial methylation pattern rather than an abrupt transition at approximately −750 bp. The two most upstream probes, cg26003280 (−1396 bp) and cg25686125 (−854 bp), were highly methylated (mean β = 0.958 and 0.918, respectively). cg26014066 at −769 bp showed an intermediate mean methylation level (β = 0.595), and the probes from −750 to −342 bp were also intermediately methylated (mean β approximately 0.17–0.24). In contrast, CpGs from −259 to +263 bp showed low absolute methylation (mean β approximately 0.019–0.047). Thus, the transition to the low-methylation TSS-proximal state occurred between the assayed positions at −342 and −259 bp, i.e., at approximately −300 bp. This methylation transition is distinct from the operational −280 bp boundary used for cross-dataset spatial integration.

Tumor-associated methylation changes were concentrated at a limited number of CpGs rather than extending uniformly across the distal promoter. In the all-sample comparison, the largest decrease was observed at cg26014066 (−769 bp; Δβ = −0.1966, 95% CI −0.2368 to −0.1556). To reduce sensitivity to cohort composition and to perform inference on M-values, tumor-normal differences were additionally tested in the 50 matched patient pairs. Three CpGs remained significant after Benjamini–Hochberg correction: cg25686125 (−854 bp; mean paired Δβ = −0.0254, 95% CI −0.0408 to −0.0118, *q* = 7.95 × 10^−4^), cg26014066 (−769 bp; Δβ = −0.1724, 95% CI −0.2185 to −0.1258, *q* = 9.62 × 10^−7^), and cg11189761 (−750 bp; Δβ = −0.0646, 95% CI −0.0909 to −0.0389, *q* = 7.18 × 10^−5^). The largest and most reproducible methylation decrease therefore occurred at cg26014066.

CpGs within the TSS-proximal region remained at low absolute methylation levels in both normal tissue and primary tumors. However, small tumor–normal differences were detected at individual sites. In the group-wise β-value analysis, small differences reached FDR significance at cg12180028 (−259 bp), cg20878850 (−178 bp), cg25085925 (−172 bp), and cg16679837 (+137 bp). Their absolute effect sizes were minimal (|Δβ| ≤ 0.0039), and none remained significant after FDR correction in the paired M-value analysis. For example, cg16679837 showed a group-wise Δβ of +0.0017, whereas its paired Δβ was −0.0003. These results indicate that low absolute proximal methylation is compatible with statistically detectable but very small group differences and provide no evidence for substantial tumor-associated hypermethylation around the TSS.

Taken together, primary tumor transformation in TCGA-PRAD was associated with localized methylation loss centered on the −854 to −750 bp region, with the dominant effect at cg26014066, rather than with a uniform change across the entire distal promoter. The TSS-proximal region remained largely unmethylated in absolute terms, and the small group-wise differences observed at several proximal CpGs were not reproduced as FDR-significant effects in the paired M-value analysis.

**Table 3:** TCGA-PRAD tumor-normal methylation differences across 15 AR promoter CpGs.

CpG	Position Relative to TSS, bp	Normal Mean β	Tumor Mean β	Δβ (All Samples)	95% CI for Δβ	Welch β *p*	BH *q*	Paired Δβ	Paired M *p*	Paired M BH *q*
cg26003280	−1396	0.9577	0.9467	−0.0110	−0.0132 to −0.0089	1.17 × 10^−19^	8.75 × 10^−19^	+0.0002	0.711	0.847
cg25686125	−854	0.9178	0.8623	−0.0555	−0.0648 to −0.0460	2.73 × 10^−21^	4.09 × 10^−20^	−0.0254	1.59 × 10^−4^	7.95 × 10^−4^
cg26014066	−769	0.5954	0.3988	−0.1966	−0.2368 to −0.1556	1.28 × 10^−13^	6.40 × 10^−13^	−0.1724	6.41 × 10^−8^	9.62 × 10^−7^
cg11189761	−750	0.2407	0.1709	−0.0698	−0.0955 to −0.0459	4.68 × 10^−7^	1.76 × 10^−6^	−0.0646	9.57 × 10^−6^	7.18 × 10^−5^
cg04755662	−504	0.1895	0.1793	−0.0103	−0.0267 to 0.0064	0.226	0.260	+0.0028	0.888	0.903
cg14807303	−418	0.1723	0.1470	−0.0253	−0.0459 to −0.0056	0.016	0.029	−0.0137	0.330	0.582
cg12156154	−416	0.1667	0.1426	−0.0242	−0.0426 to −0.0067	0.013	0.028	−0.0145	0.134	0.427
cg12294334	−362	0.1824	0.1701	−0.0123	−0.0249 to 0.0003	0.072	0.090	+0.0013	0.903	0.903
cg10164191	−342	0.1688	0.1716	+0.0029	−0.0142 to 0.0201	0.754	0.808	+0.0184	0.349	0.582
cg12180028	−259	0.0447	0.0420	−0.0026	−0.0049 to −0.0005	0.027	0.040	−0.0009	0.275	0.582
cg20878850	−178	0.0469	0.0431	−0.0039	−0.0066 to −0.0011	0.010	0.026	−0.0022	0.251	0.582
cg25085925	−172	0.0187	0.0199	+0.0012	0.0002 to 0.0022	0.025	0.040	−0.0008	0.142	0.427
cg08063601	+79	0.0281	0.0279	−0.0002	−0.0024 to 0.0018	0.833	0.833	−0.0008	0.518	0.706
cg16679837	+137	0.0209	0.0227	+0.0017	0.0009 to 0.0026	3.82 × 10^−4^	0.0011	−0.0003	0.405	0.607
cg05786601	+263	0.0241	0.0223	−0.0018	−0.0034 to −0.0002	0.042	0.057	−0.0000	0.734	0.847

Note: Group-wise values use all available tumor and normal samples and report mean β differences (tumor − normal), 95% bootstrap confidence intervals, and Welch’s two-sample tests on β-values with Benjamini–Hochberg correction across 15 CpGs. The paired analysis uses 50 matched tumor-normal patient pairs. Hypothesis testing in the paired analysis was performed on M-values using two-sided paired *t*-tests with Benjamini–Hochberg correction; paired Δβ values are reported as effect sizes on the β scale. cg25686125 had one missing tumor β-value in the all-sample comparison (*n* = 497); all other CpGs used 498 tumors and 50 normal samples. Abbreviations: BH, Benjamini–Hochberg; CI, confidence interval; CpG, cytosine–phosphate–guanine dinucleotide; TCGA-PRAD, The Cancer Genome Atlas Prostate Adenocarcinoma; TSS, transcription start site; bp, base pairs.

To directly assess the relationship between AR promoter methylation and AR expression, methylation and RNA data were matched at the sample level in TCGA-PRAD. Matched data were available for 497 primary tumors and 35 normal samples. In tumors, four of the 15 CpGs remained associated with AR mRNA after correction for multiple testing, but all correlations were weak (absolute Spearman ρ ≤ 0.190; Supplementary Table S1): cg26003280 (ρ = 0.136, *q* = 0.0174), cg12294334 (ρ = 0.119, *q* = 0.0288), cg25085925 (ρ = −0.131, *q* = 0.0178), and cg16679837 (ρ = −0.190, *q* = 0.000301).

For cg26014066, which showed the largest tumor-associated methylation decrease, the association with AR mRNA was weak and did not remain significant after FDR correction (ρ = −0.089, *p* = 0.0465, *q* = 0.1395), and a single-predictor linear model explained 1.20% of AR mRNA variance. The strongest individual correlation, at cg16679837, explained only 2.10% of the variance. A joint model containing all 15 CpGs explained 8.38% of AR mRNA variance in the training data (adjusted *R*^2^ = 5.51%), whereas its mean *R*^2^ across 20 repeats of five-fold cross-validation was approximately zero (−0.00114). No CpG association survived FDR correction in normal samples or in the paired methylation–RNA change analysis. These results indicate that promoter methylation alone has limited explanatory value for AR expression in primary tumors.

#### 2.3.2. Comparison of AR Promoter Methylation in C4-2B and NCI-H660 Cell Models

To compare AR-positive castration-resistant and AR-negative neuroendocrine states, data from GSE158598 generated using the Illumina Infinium MethylationEPIC platform were analyzed. The AR-positive designation of C4-2B and the AR-negative neuroendocrine designation of NCI-H660 were adopted from the corresponding source literature and were not independently reassessed in the present study. The dataset included one sample each of the C4-2B and NCI-H660 cell lines; of the 15 CpG positions examined in TCGA-PRAD, 13 were represented on the EPIC array.

C4-2B is an adherent epithelial-like prostate adenocarcinoma subline derived from LNCaP cells. C4-2B cells retain transcriptionally active AR signaling and can proliferate under androgen-depleted conditions. Accordingly, this cell line is commonly used as a model of AR-positive CRPC. NCI-H660 is a neuroendocrine small-cell prostate cancer cell line. These cells lack AR and PSA expression and express neuroendocrine markers, supporting their use as a model of AR-negative neuroendocrine prostate cancer.

**Figure 3:**
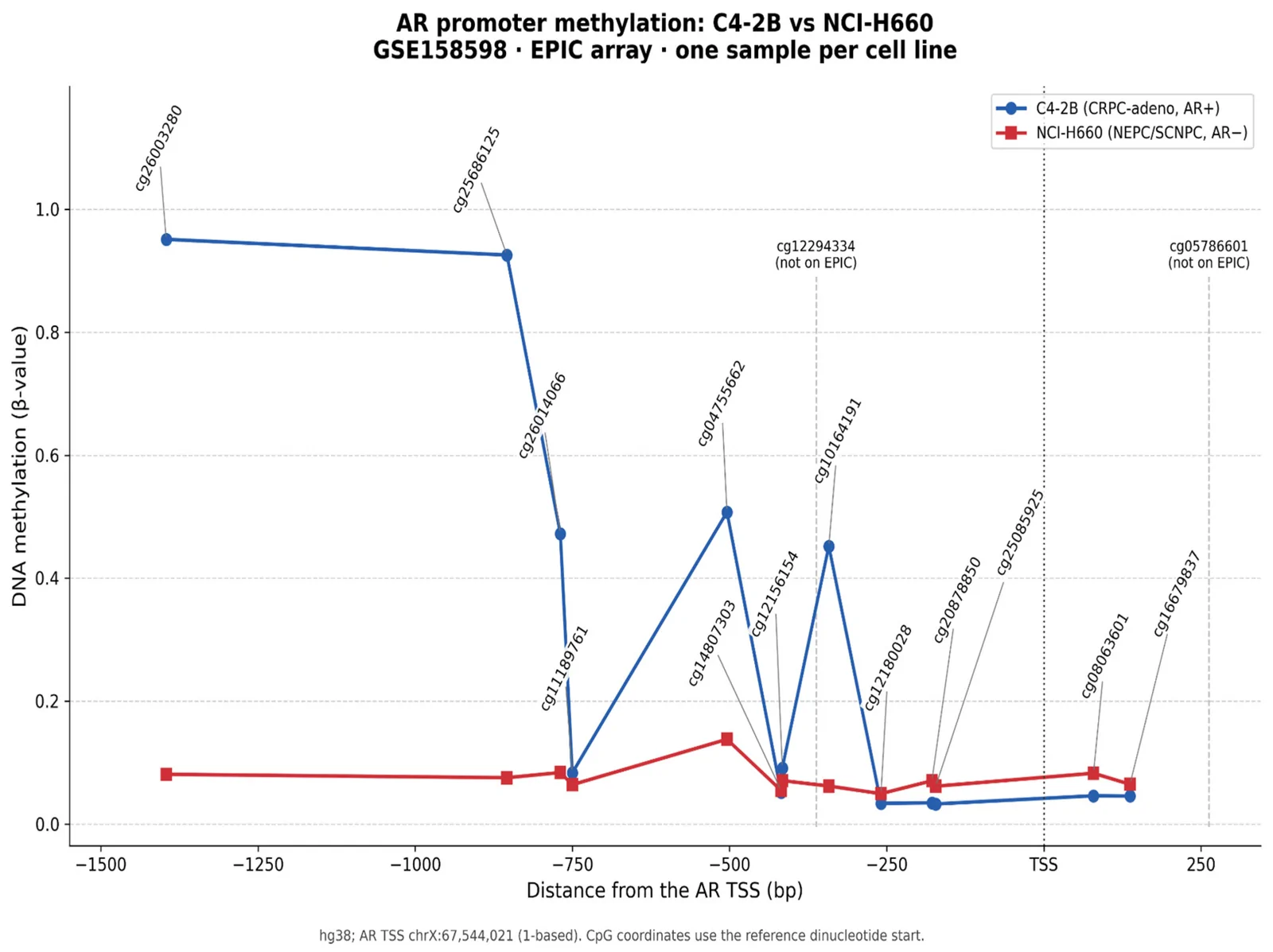
Methylation of the AR promoter in C4-2B (CRPC-adeno, AR+) and NCI-H660 (NEPC/SCNPC, AR−) cells based on GSE158598 data (Illumina EPIC). The *x*-axis represents the distance of CpG loci from the AR TSS, and the *y*-axis shows methylation β-values (0–1). Blue circles denote C4-2B cells, and red squares denote NCI-H660 cells. The dotted vertical line marks the TSS; the light gray dashed lines identify CpG loci absent from the EPIC array. Abbreviations: CpG, cytosine–phosphate–guanine dinucleotide; CRPC-adeno, castration-resistant prostate adenocarcinoma; EPIC, Infinium MethylationEPIC array; NEPC, neuroendocrine prostate cancer; SCNPC, small-cell/neuroendocrine prostate cancer; TSS, transcription start site; bp, base pairs.

The comparison of AR promoter methylation between these cell lines is shown in Figure 3.

The most pronounced methylation differences were concentrated in the distal portion of the promoter. At cg26003280 (−1396 bp), β-values were 0.951 in C4-2B and 0.081 in NCI-H660, whereas at cg25686125 (−854 bp) they were 0.925 and 0.076, respectively. Lower methylation in NCI-H660 was also observed at cg26014066 (−769 bp), cg04755662 (−504 bp), and cg10164191 (−342 bp). Thus, NCI-H660 cells exhibited marked loss of methylation across a broad distal promoter segment compared with C4-2B cells.

Proximal CpG sites near the TSS remained hypomethylated in both cell lines, predominantly within the β = 0.03–0.08 range. Therefore, the absence of AR expression in NCI-H660 cells was not accompanied by hypermethylation of the proximal promoter. Instead, the AR-negative state in this model was associated with lower methylation of the distal region than that observed in AR-positive C4-2B cells.

This finding is inconsistent with a simplified model in which promoter hypomethylation necessarily corresponds to gene activation. Rather, distal demethylation likely reflects differences in the epigenetic state and cellular identity of the two models rather than direct activation or silencing of AR transcription.

#### 2.3.3. AR Promoter Methylation Across Phenotypic States of Metastatic CRPC in the WCDT Cohort

To assess methylation of the AR promoter across phenotypically distinct states of metastatic castration-resistant prostate cancer (mCRPC), published whole-genome bisulfite sequencing (WGBS) results from the West Coast Dream Team (WCDT) cohort were examined. Tumor phenotypes were defined in the source study according to AR and neuroendocrine (NE) transcriptional program activity and were used here without reclassification: AR-high prostate cancer (ARPC; AR+/NE−; *n* = 40), AR-low prostate cancer (ARLPC; AR-low/NE−; *n* = 21), amphicrine prostate cancer (AMPC; AR+/NE+; *n* = 4), double-negative prostate cancer (DNPC; AR−/NE−; *n* = 2), and small-cell/neuroendocrine prostate cancer (SCNPC/NEPC; AR−/NE+; *n* = 3).

**Figure 4:**
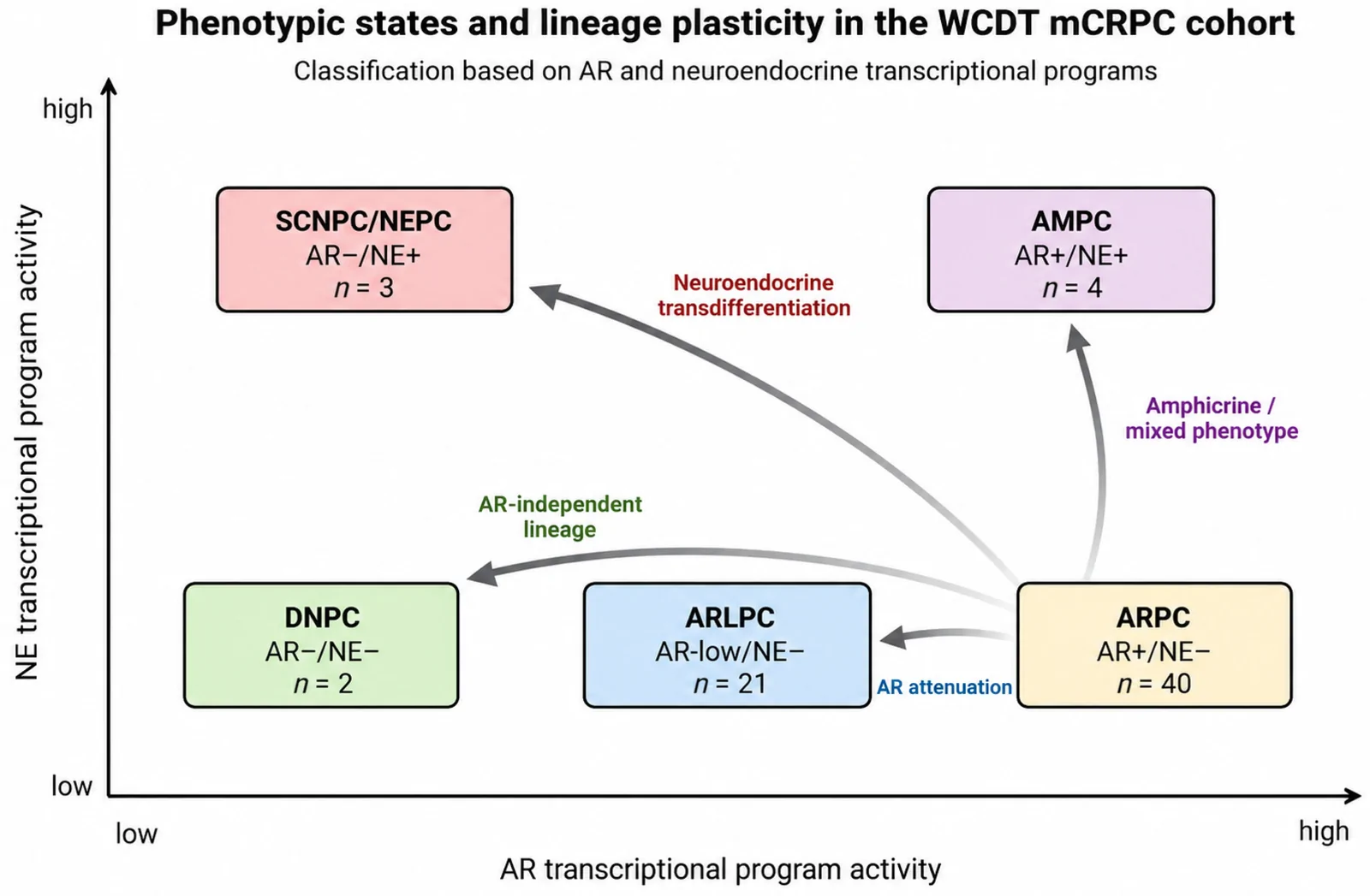
Hypothetical literature-supported model of phenotypic diversification from an AR-active mCRPC state represented by ARPC toward ARLPC, AMPC, DNPC, and SCNPC/NEPC. The branching framework summarizes lineage-plasticity relationships described in clinical, PDX, and experimental studies [44]; the neuroendocrine branch is additionally supported by longitudinal genomic evidence consistent with divergent clonal evolution [45]. Sample numbers indicate the WCDT WGBS groups analyzed here. The branching connections indicate hypothetical phenotypic relationships and do not represent longitudinally observed transitions in the WCDT cohort. Abbreviations: AMPC, amphicrine prostate cancer; ARLPC, AR-low prostate cancer; ARPC, AR-high prostate cancer; DNPC, double-negative prostate cancer; mCRPC, metastatic castration-resistant prostate cancer; NE, neuroendocrine; NEPC, neuroendocrine prostate cancer; PDX, patient-derived xenograft; SCNPC, small-cell/neuroendocrine prostate cancer; WCDT, West Coast Dream Team; WGBS, whole-genome bisulfite sequencing.

Within this framework, ARPC represents the AR-active mCRPC state. Phenotypic profiling of clinical metastases, patient-derived xenografts, and experimental models has supported diversification from ARPC toward AR-low, amphicrine, double-negative, and neuroendocrine states under AR-pathway suppression [44], while longitudinal genomic analyses provide direct evidence consistent with divergent clonal evolution from castration-resistant adenocarcinoma toward treatment-emergent neuroendocrine prostate cancer [45]. ARPC was therefore used as the biological reference state for examining AR-promoter methylation across these divergent phenotypic branches (Figure 4).

**Figure 5:**
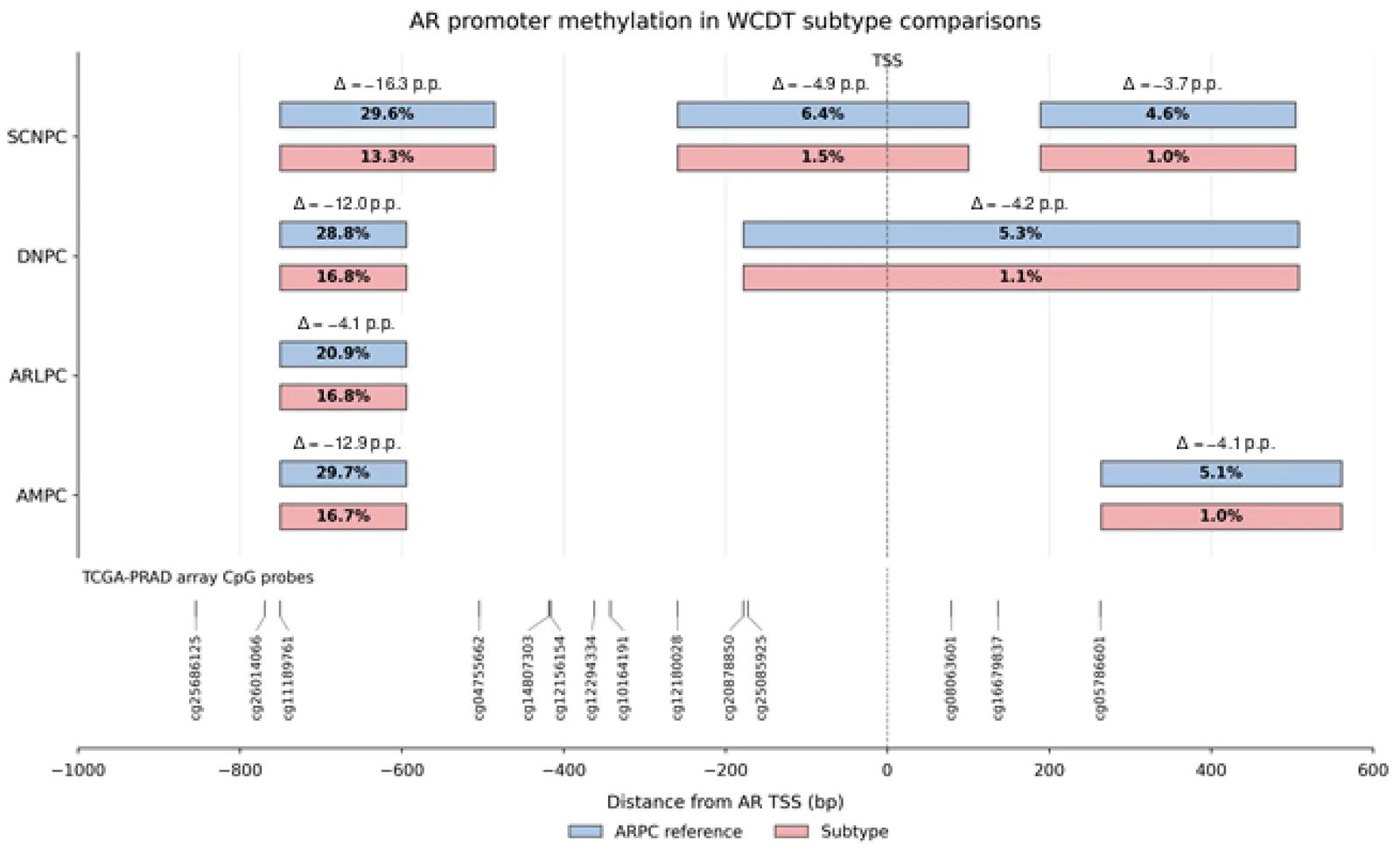
Source-reported WCDT regional records overlapping the AR promoter and their relationship to TCGA-PRAD array CpG positions. Blue bars show the regional methylation estimate for the contrast-specific ARPC reference and pink bars the corresponding subtype estimate. Coordinates are relative to the AR TSS; Δ is expressed in percentage points (subtype − ARPC). Each subtype was compared independently with ARPC. The array CpG track is shown for spatial reference only. A lower regional methylation estimate is observed in the compared subtype for every promoter-overlapping WCDT record shown. Abbreviations: AMPC, amphicrine prostate cancer; ARLPC, AR-low prostate cancer; ARPC, AR-high prostate cancer; CpG, cytosine–phosphate–guanine dinucleotide; DNPC, double-negative prostate cancer; SCNPC, small-cell/neuroendocrine prostate cancer; TCGA-PRAD, The Cancer Genome Atlas Prostate Adenocarcinoma; TSS, transcription start site; WCDT, West Coast Dream Team; bp, base pairs; p.p., percentage points.

Each subtype was compared independently with ARPC in the published DSS analysis. Eight source-reported regional records overlapped the analyzed AR promoter window. Their exact coordinates, widths, unique reference CpG counts, and source areaStat values are summarized in Table 4; regional methylation estimates and absolute differences are shown in Figure 5. Because the regions were defined separately for each subtype-versus-ARPC contrast, their boundaries and corresponding ARPC regional methylation estimates are contrast-specific and were not treated as measurements of a single common DMR across all phenotypic states.

Across all eight promoter-overlapping WCDT records, regional methylation was lower in the investigated phenotype than in its contrast-specific ARPC reference. In the recurrent distal region beginning at approximately −750 bp, methylation changed from 20.85% to 16.76% in ARLPC (Δ = −4.1 p.p.), from 29.65% to 16.74% in AMPC (Δ = −12.9 p.p.), from 28.76% to 16.75% in DNPC (Δ = −12.0 p.p.), and from 29.61% to 13.35% in SCNPC (Δ = −16.3 p.p.). Additional records spanning the TSS-proximal or downstream region also showed lower subtype estimates, although the corresponding ARPC regions were already weakly methylated (4.63–6.42%). The directionally concordant decrease across AR-low, amphicrine, double-negative, and neuroendocrine states therefore supports a general shift toward reduced AR-promoter methylation across divergent lineage-plasticity states relative to the AR-active mCRPC state. Because DMR boundaries were contrast-specific, this pattern indicates a shared direction of methylation remodeling rather than an ordered quantitative gradient among phenotypes.

Nevertheless, the concordant direction of the WCDT changes across all promoter-overlapping records identifies reduced regional AR-promoter methylation as a recurrent feature of phenotypic diversification from the AR-active mCRPC state. Interpretation of the magnitude of this remodeling is limited by the small DNPC (*n* = 2) and SCNPC (*n* = 3) groups and by the absence of individual sample-level WGBS methylation values in the released aggregate output; therefore, no patient-level comparison of effect magnitude or subtype ordering was performed.

### 2.4. ChIP-Seq Analysis of TF Binding Within the AR Promoter

A total of 1121 successfully queried hg38 ChIP-seq datasets were analyzed for 10 TFs at a threshold of *q* < 1 × 10^−5^. To assess the reproducibility of binding within the AR promoter, the results were organized by TF and biological model, considering both the presence of a promoter peak and its overlap with the selected JASPAR motifs (Table 5).

Among the 10 TFs analyzed, seven showed at least one ChIP-seq peak within the AR promoter: CTCF, FOXA1, HOXB13, AR, NR3C1/GR, E2F1, and ERG. After restriction of the primary motif–ChIP analysis to strict-tier JASPAR motifs (*p* < 1 × 10^−4^), same-TF ChIP-seq overlap remained for FOXA1 and E2F1. The strict-tier FOXA1 motif at −685 bp was overlapped in 89 of 272 successfully queried FOXA1 datasets (32.7%); circular permutation yielded an empirical *p* = 0.041. The strict-tier E2F1 motif at −264 bp was overlapped in 2 of 11 datasets (18.2%), but this did not exceed the permutation expectation (empirical *p* = 0.349). The CTCF motif at −867 bp and the proximal ERG motifs belonged to the supportive tier and were therefore retained as descriptive motif–ChIP overlaps rather than primary strict-tier overlaps. ChIP-seq peaks that did not overlap the corresponding selected motifs were analyzed separately (Table 6).

Integration of the ChIP-seq results revealed pronounced context-dependent organization of the *AR* regulatory region. The most reproducible distal element was the FOXA1 module at −751 to −678 bp, containing the −751, −717, and −685 bp motifs, which were overlapped by a common broad ChIP-seq peak in FOXA1-positive datasets. FOXA1 promoter peaks were detected in 31/123 LNCaP datasets and 33/49 VCaP datasets; in VCaP, 31/49 datasets directly overlapped the −751/−717/−685 bp motif cluster. In contrast, no detectable FOXA1 occupancy within the analyzed promoter interval was observed in any of the six available C4-2B datasets.

VCaP showed reproducible ERG occupancy within the proximal AR promoter. The corresponding ERG motifs were supportive-tier predictions and are therefore interpreted as descriptive motif–ChIP overlaps rather than primary strict-tier motif support. In the same model, the recurrent proximal AR ChIP-seq signal spanned approximately −308 to +266 bp and did not coincide with the predicted high-affinity AREs, which were located farther upstream. Quantitative reanalysis of four VCaP AR ChIP-seq datasets from GSE211138 (two DMSO+DHT and two ML792+DHT datasets; Table 7) showed Input-normalized AR enrichment of 4.40–10.43 within −280 to +100 bp and 4.46–9.08 across the broader −308 to +266 bp interval, compared with 1.56–3.71 across the full −2000 to +500 bp promoter. A VCaP IgG control matched to the DMSO+DHT condition showed substantially lower enrichment (IgG/Input = 1.68 within −280 to +100 bp and 1.62 within −308 to +266 bp). Thus, although a modest TSS-associated nonspecific signal was detectable, the magnitude of the proximal AR enrichment was substantially greater. Because conventional ChIP-seq cannot distinguish direct sequence-specific AR–DNA binding from indirect or complex-associated chromatin occupancy, this motif-free proximal signal is interpreted as AR-associated enrichment rather than evidence of direct AR binding. Its spatial overlap with reproducible ERG occupancy raises the hypothesis that AR and ERG may participate in a common proximal regulatory configuration; however, demonstration of simultaneous locus-specific occupancy would require orthogonal validation such as sequential ChIP/re-ChIP.

Quantitative AR/Input enrichment across the four AR datasets ranged from 4.40 to 10.43 in the −280 to +100 bp interval, from 4.46 to 9.08 in the recurrent −308 to +266 bp proximal AR peak, and from 1.56 to 3.71 across the full −2000 to +500 bp promoter. The DMSO+DHT IgG/Input benchmark was 1.68 in the −280 to +100 bp interval and 1.62 in the −308 to +266 bp interval. Because the IgG dataset is DMSO+DHT, the most direct AR-versus-IgG comparison is with the two DMSO+DHT AR replicates; the two ML792+DHT AR datasets provide additional evidence for reproducibility of proximal AR enrichment under a distinct perturbation condition.

**Figure 6:**
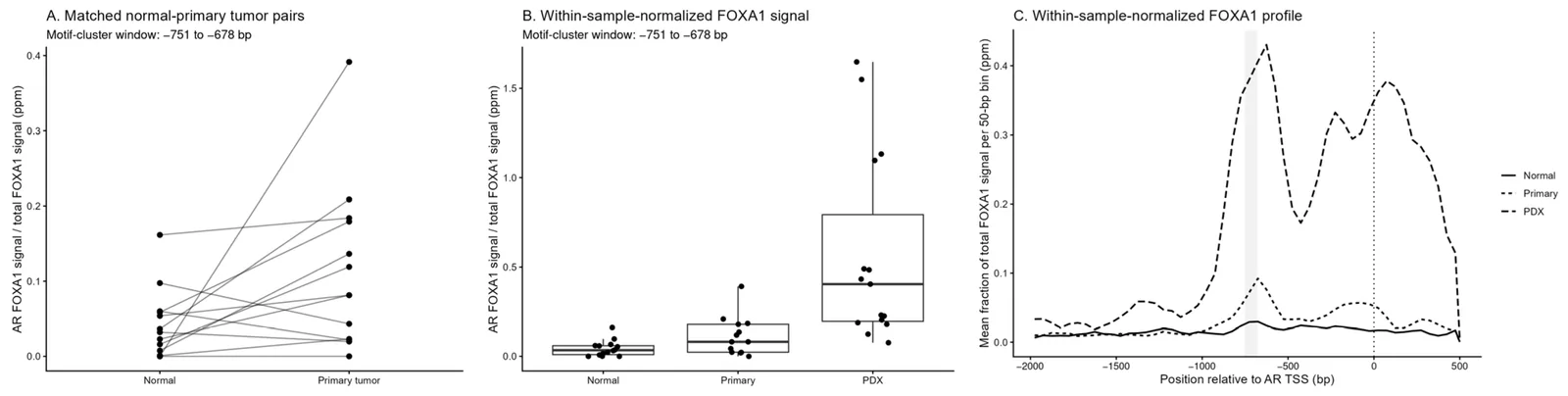
Quantitative assessment of FOXA1 signal at the distal AR promoter module (−751 to −678 bp). (**A**) Paired comparison between normal tissue and primary tumor in 13 patients. (**B**) Distribution in normal tissue (*n* = 14), primary tumors (*n* = 13), and mCRPC-derived LuCaP PDXs (*n* = 15). (**C**) Mean FOXA1 signal profile across the AR promoter; the −751 to −678 bp analysis window is shaded. Cross-series statistical comparisons reported in the text are based on within-sample-normalized FOXA1 signal. In panel C, the vertical dotted line marks the AR TSS (0 bp); solid, dotted, and dashed curves represent normal tissue, primary tumors, and PDXs, respectively. Abbreviations: mCRPC, metastatic castration-resistant prostate cancer; PDX, patient-derived xenograft; ppm, parts per million of total FOXA1 signal; TSS, transcription start site; bp, base pairs.

Quantitative analysis of GSE130408 data supported increased FOXA1 signal within the distal module from normal prostate tissue to primary tumor. In 13 matched normal–primary tumor pairs, within-sample-normalized FOXA1 signal was higher in primary tumors in both the broader −814 to −614 bp interval (10/13 patients; *p* = 0.019) and the narrower −751 to −678 bp FOXA1 motif-cluster interval (9/13 patients; *p* = 0.034). After the same normalization, FOXA1 signal within the −751 to −678 bp interval remained higher in mCRPC-derived PDXs than in normal prostate tissue (*p* = 8.50 × 10^−6^) or primary tumors (*p* = 5.05 × 10^−4^). The normalized promoter profile localized this relative enrichment to the −751/−717/−685 bp FOXA1 motif cluster (Figure 6).

Because clinical tissues and PDXs differ in cellular composition and experimental context, the PDX comparison is interpreted as relative enrichment of AR-associated FOXA1 signal rather than as a direct measure of absolute FOXA1 occupancy.

In 31 matched normal–primary tumor pairs, H3K27ac did not show a statistically significant systematic change in either interval. The mean paired difference was +0.0270 in the distal −751 to −678 bp interval (95% bootstrap CI, −0.0096 to +0.0652; two-sided exact Wilcoxon signed-rank *p* = 0.294) and +0.0684 in the proximal −280 to +100 bp interval (95% bootstrap CI, −0.0101 to +0.1531; *p* = 0.169). Thus, the paired clinical data did not support a cohort-wide increase in H3K27ac in either region.

**Figure 7:**
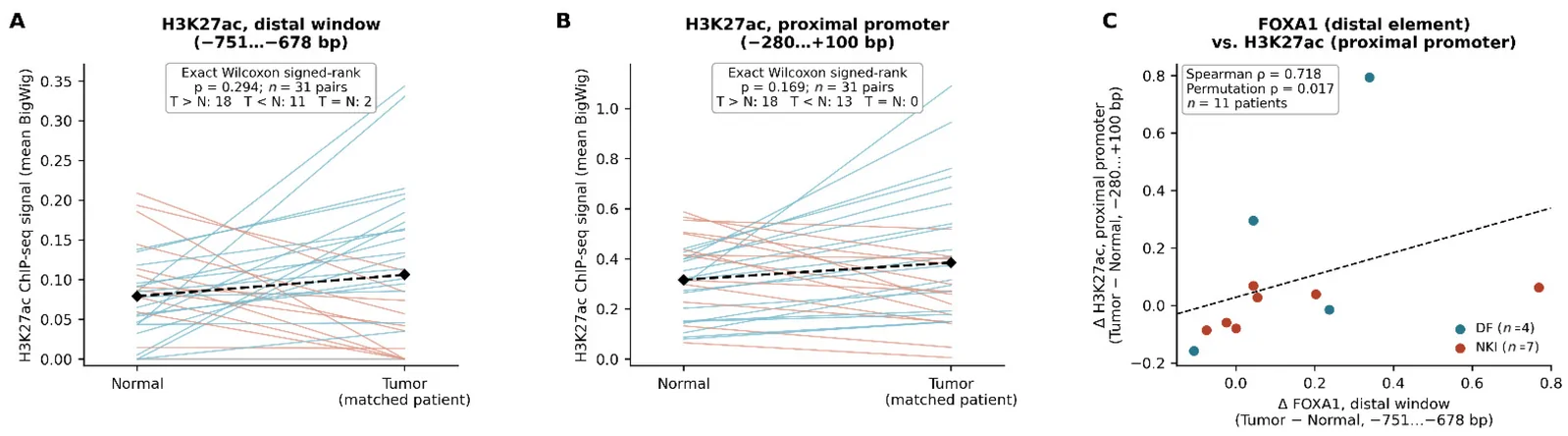
Paired H3K27ac changes and their relationship with FOXA1 remodeling within the AR promoter. (**A**) Paired H3K27ac signal in normal tissue and primary tumor within the distal −751 to −678 bp interval. (**B**) The corresponding paired comparison within the proximal −280 to +100 bp interval. Panels (**A**,**B**) include 31 matched patient pairs (24 DF and seven NKI). (**C**) Association between tumor-associated changes in distal FOXA1 signal and proximal H3K27ac in the 11 patients with paired measurements of both marks. Teal lines/points denote DF samples, and orange-red lines/points denote NKI samples. In panels (**A**,**B**), the black dashed line connecting black diamonds shows the overall paired mean trend; T and *N* denote tumor and normal signals, respectively. In panel (**C**), the dashed line shows the fitted association trend. DF, Dana-Farber Cancer Institute; NKI, Netherlands Cancer Institute. H3K27ac, acetylation of histone H3 at lysine 27; ChIP-seq, chromatin immunoprecipitation followed by sequencing; bp, base pairs.

Despite the absence of a significant cohort-wide increase in H3K27ac, tumor-associated changes in FOXA1 and H3K27ac showed evidence of coordinated inter-individual remodeling. Among the 11 patients for whom paired measurements of both marks were available, the change in FOXA1 signal within the distal −751 to −678 bp module was positively associated with the change in H3K27ac within the proximal −280 to +100 bp region (Spearman ρ = 0.718; permutation *p* = 0.017; 95% bootstrap CI, 0.094 to 0.971; Figure 7C). The corresponding association with H3K27ac within the same distal interval was weaker (ρ = 0.382; permutation *p* = 0.247; 95% bootstrap CI, −0.346 to 0.850). However, the difference between the two correlations was not statistically established (Δρ = 0.336; 95% bootstrap CI, −0.369 to 1.019; Williams-type test *p* = 0.298). Accordingly, the proximal association should be regarded as exploratory evidence of coordinated FOXA1–H3K27ac remodeling rather than proof of region-specific coupling.

The positive association was not driven by a single observation: leave-one-out sensitivity analysis yielded Spearman ρ values ranging from 0.624 to 0.806. Exclusion of the highest-leverage observation still resulted in a positive association (ρ = 0.661; nominal *p* = 0.038). Although the direction and magnitude of the association remained relatively stable, leave-one-out nominal *p*-values ranged from 0.0049 to 0.0537, indicating some sensitivity of statistical significance to sample composition, as expected given the small sample size.

The cell-line data further indicate that increased activity of the FOXA1 module is not the only configuration compatible with an AR-positive state. C4-2B retains an AR-positive castration-resistant phenotype despite the absence of detectable FOXA1 occupancy within the examined distal module. AR-negative DU145 represents another configuration, with no FOXA1 promoter peak detected in any of five datasets and no AR binding detected in any of 11 datasets. Thus, the analysis identifies multiple context-dependent configurations of *AR* regulatory architecture that differ in their combinations of FOXA1, ERG, and AR occupancy.

### 2.5. Active and Repressive Chromatin States of the AR Promoter

To assess active chromatin at the AR promoter, 516 H3K27ac ChIP-seq datasets were screened for peaks across the −2000 to +500 bp interval relative to the TSS (Table 8).

Overall, H3K27ac peaks were detected in 145/516 (28.1%) datasets. The distribution of H3K27ac differed substantially across cell models. The most reproducible enrichment was observed in VCaP, where a promoter H3K27ac peak was present in all 27/27 datasets, as well as in C4-2B (4/4) and its enzalutamide-resistant derivative C4-2B-ER (2/2). H3K27ac was detected in 6/14 (42.9%) C4-2 datasets, 56/146 (38.4%) LNCaP datasets, and only 9/62 (14.5%) 22Rv1 datasets. In contrast, no H3K27ac promoter peaks were detected in AR-negative DU145 across all eight datasets, whereas PC-3 showed promoter H3K27ac in only 1/37 (2.7%) datasets. Thus, an active H3K27ac promoter state was generally associated with AR-positive models, although its reproducibility was strongly context-dependent.

H3K27ac showed particularly pronounced spatial localization within the proximal promoter. Of the 145 H3K27ac-positive datasets, 137 contained a peak overlapping the −280 to +100 bp region. This was observed in 26/27 positive VCaP datasets, 4/4 C4-2B datasets, 6/6 C4-2 datasets, 50/56 LNCaP datasets, and 9/9 22Rv1 datasets. None of the called H3K27ac peaks directly overlapped the distal FOXA1 module at −751 to −678 bp.

In the 11 paired cases, changes in distal FOXA1 signal were positively associated with proximal H3K27ac changes, whereas the corresponding distal association was weaker. However, the difference between the two correlations was not statistically significant. We therefore interpret this finding as exploratory evidence of coordinated FOXA1–H3K27ac remodeling rather than region-specific coupling.

A similar pattern was observed in clinical samples. H3K27ac promoter peaks were detected in 35/152 (23.0%) primary tumor datasets compared with 2/38 (5.3%) morphologically normal tissue datasets; 34 of the 35 H3K27ac-positive tumor datasets contained a peak within the proximal −280 to +100 bp region. In liver mCRPC metastases, H3K27ac was detected in 2/3 datasets and was likewise concentrated predominantly around the TSS. These observations are consistent with strengthening of an active *AR* promoter configuration during tumor progression, although frequency differences across aggregated ChIP-seq datasets should be interpreted in light of the heterogeneity of the underlying experiments.

**Figure 8:**
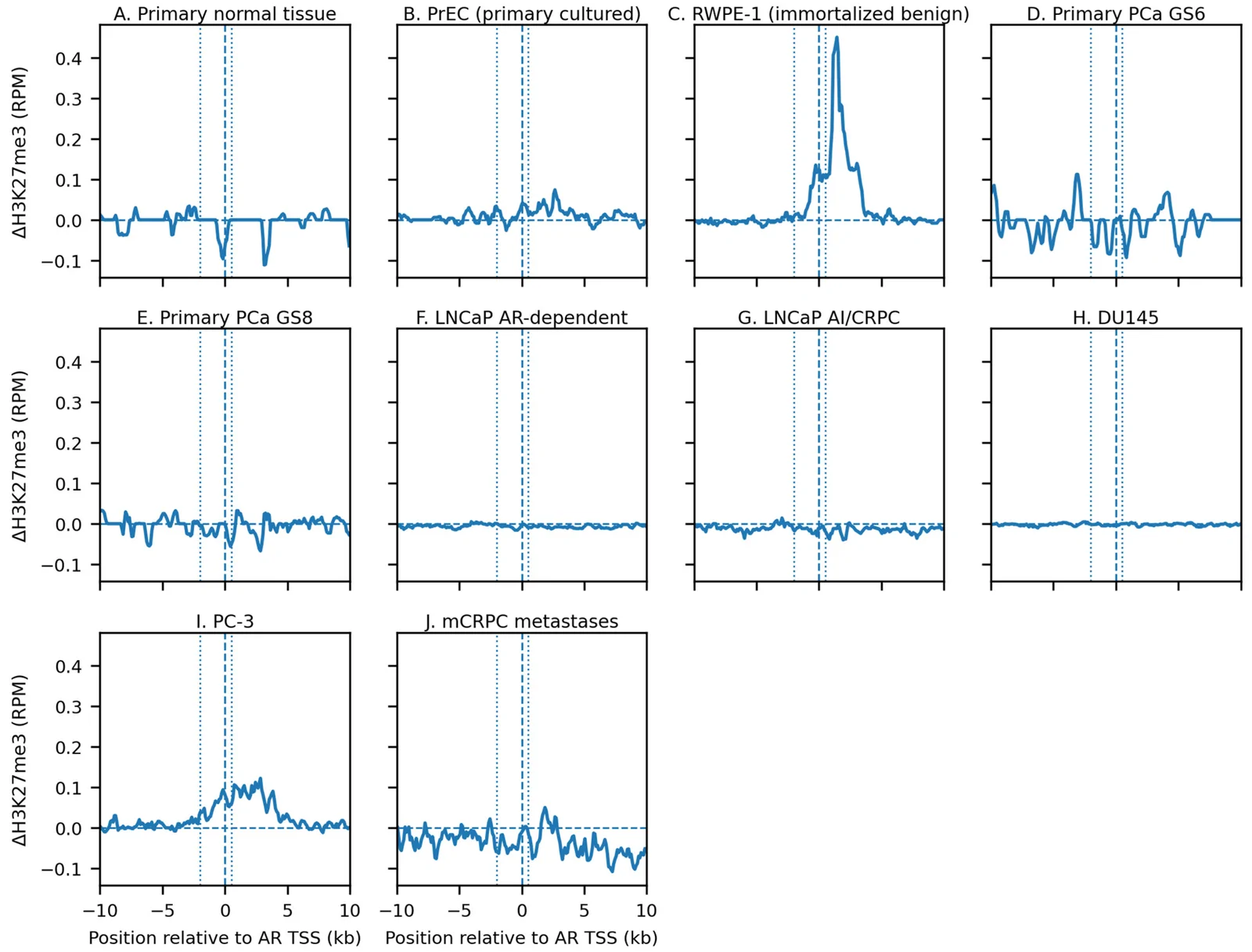
Input-corrected H3K27me3 profiles across the *AR* locus. ΔH3K27me3 = RPM_ChIP_ − RPM_Input_ was calculated in 100-bp bins and smoothed over a centered 500-bp window. Group profiles for the 31 valid matched pairs are study-first medians; RWPE-1 (GSE63094) is shown separately using two H3K27me3 ChIP replicates and one matched Input. Panels show (**A**) primary normal prostate tissue; (**B**) PrEC; (**C**) RWPE-1; (**D**) primary PCa with GS6; (**E**) primary PCa with GS8; (**F**) AR-dependent LNCaP; (**G**) androgen-independent/CRPC LNCaP; (**H**) DU145; (**I**) PC-3; and (**J**) mCRPC metastases. All panels show ±10 kb relative to the *AR* TSS with a common *y*-axis scale. Dotted lines mark −2 kb and +0.5 kb, and the vertical dashed line marks the TSS. The horizontal dashed line indicates zero Input-corrected signal. Positive values indicate ChIP signal above matched Input. Cross-study ΔRPM values are descriptive and are not spike-in-calibrated. Abbreviations: AI, androgen-independent; ChIP, chromatin immunoprecipitation; CRPC, castration-resistant prostate cancer; GS, Gleason score; H3K27me3, trimethylation of histone H3 at lysine 27; mCRPC, metastatic CRPC; PCa, prostate cancer; PrEC, primary prostate epithelial cells; RPM, reads per million; TSS, transcription start site; bp, base pairs; kb, kilobases.

Because H3K27me3 is a broad histone mark, quantitative H3K27me3 analysis was performed for 31 technically valid matched ChIP/Input pairs representing primary normal prostate tissue, PrEC, primary prostate cancer (GS6 and GS8), LNCaP AR-dependent and androgen-independent/CRPC states, DU145, PC-3, and mCRPC metastases. For each pair, H3K27me3 and Input signals were compared in 100-bp bins across ±10 kb from the *AR* TSS. The primary Input-corrected signal was defined as ΔH3K27me3 = RPM_ChIP_ − RPM_Input_ and smoothed over a centered 500-bp window. RWPE-1 (GSE63094) was analyzed separately as an independent benign immortalized epithelial reference using two H3K27me3 replicates and a matched Input. The resulting profiles are shown in Figure 8.

The quantitative profiles did not show a monotonic increase or decrease in H3K27me3 across the progression sequence. Primary normal tissue and primary PCa groups showed near-zero or locally negative Input-corrected signal around the TSS, and both AR-dependent and androgen-independent LNCaP states remained low. DU145 likewise lacked a strong TSS-centered H3K27me3 configuration, and the analyzed mCRPC metastases showed no reproducible positive domain. In contrast, PC-3 displayed a broad and reproducible TSS-centered H3K27me3-enriched configuration across three independent study blocks. Thus, loss of *AR* expression was not uniformly associated with acquisition of H3K27me3: PC-3 and DU145 represented distinct AR-negative chromatin states.

Benign cultured models also differed from primary normal tissue. PrEC showed a modest TSS-centered positive ΔH3K27me3 profile, whereas RWPE-1 showed a pronounced TSS-centered enrichment in the independent GSE63094 dataset. Because PrEC and RWPE-1 are cultured epithelial models rather than intact normal prostate tissue, they were not pooled with primary normal samples. Window-level group summaries are presented in Table 9.

Across predefined windows, PC-3 showed positive study-first median ΔRPM values of 0.0545 in the −2/+0.5 kb promoter interval and 0.0670 across TSS ±2 kb; RWPE-1 showed values of 0.0600 and 0.1400, respectively. By contrast, DU145 remained close to zero (−0.0030 and −0.0023), and the analyzed mCRPC metastases showed negative Input-corrected values (−0.0295 and −0.0249). These values are descriptive because RPM normalization and Input subtraction do not remove all inter-study technical variation, and no spike-in calibration was available.

Together, these data show that H3K27me3 at the AR locus is context-dependent, with pronounced enrichment in PC-3 and RWPE-1 but no comparable configuration in DU145, the primary tumor groups, or the analyzed mCRPC metastases.

C4-2B represents an alternative configuration of AR regulatory architecture. This AR-positive CRPC model retains an active H3K27ac promoter state in all available datasets, with peaks concentrated predominantly within the proximal TSS-associated region, despite the absence of detectable FOXA1 binding at the distal −751 to −678 bp module. Collectively, these observations suggest an alternative active promoter configuration in C4-2B that is maintained without detectable FOXA1 occupancy at this distal module.

### 2.6. Spatial Organization of ncPRC1.1-Associated Components and H2AK119ub1 Within the AR Promoter

#### Proximal Enrichment of ncPRC1.1 Components and H2AK119ub1 in PC-3 Cells

The quantitative H3K27me3 analysis identified an extended TSS-centered H3K27me3-enriched configuration in PC-3 cells. The presence of this broad mark does not exclude additional involvement of variant PRC1 complexes in shaping the local chromatin state. In particular, KDM2B recognizes unmethylated CpG-rich sequences through its ZF-CxxC domain and contributes to recruitment of the PRC1.1 complex, which contains PCGF1 and the catalytic component RNF2/RING1B. RNF2/RING1B mediates monoubiquitination of histone H2A at Lys119, generating H2AK119ub1 [46,47]. To assess the potential involvement of this mechanism in regulation of the *AR* promoter, raw ChIP-seq data for KDM2B, PCGF1, RNF2, and H2AK119ub1 from PC-3 cells in dataset GSE131829 were analyzed [48]. Two functionally defined intervals were selected for spatial analysis. The distal region at −751 to −678 bp corresponded to the previously identified FOXA1 motif cluster containing the ChIP-supported motifs at −751, −717, and −685 bp. The proximal interval at −280 to +100 bp encompassed the region around the TSS and included the previously identified ERG-associated module containing motifs at −280, −225, −104, and −82 bp.

Within the FOXA1 motif cluster at −751 to −678 bp, no coordinated enrichment of the full set of ncPRC1.1-associated proteins and H2AK119ub1 was observed. Enrichment relative to Input was 0.825-fold for KDM2B, 2.931-fold for PCGF1, and 1.475-fold for RNF2. H2AK119ub1 showed discordant values in two independent replicates, with enrichments of 2.329-fold and 0.770-fold, respectively (Table 10). Thus, the moderate local increase in PCGF1 and in H2AK119ub1 in one replicate was not accompanied by reproducible H2AK119ub1 accumulation or coordinated enrichment of KDM2B, PCGF1, and RNF2. These findings do not support classification of the FOXA1 motif cluster as a stable local PRC1.1/H2AK119ub1-associated region.

In contrast, the proximal interval at −280 to +100 bp showed pronounced concurrent enrichment of KDM2B (4.952-fold), PCGF1 (3.957-fold), and RNF2 (2.655-fold), accompanied by marked accumulation of H2AK119ub1 in two independent replicates, with enrichments of 8.852-fold and 8.773-fold, respectively (Table 10). The coordinated presence of KDM2B, PCGF1, and RNF2 together with reproducible accumulation of the repressive histone modification H2AK119ub1 is consistent with the formation of a PRC1.1-associated chromatin state with a potentially repressive function in the proximal AR promoter region.

Collectively, these findings indicate pronounced spatial polarity of Polycomb-associated chromatin across the *AR* promoter in PC-3 cells. The PRC1.1/H2AK119ub1-associated configuration is sharply concentrated in the proximal promoter around the TSS, whereas the H3K27me3 enrichment extends across a broader TSS-centered region. The marked and reproducible accumulation of H2AK119ub1 is therefore superimposed on, rather than substituted for, an H3K27me3-enriched chromatin background. This layered configuration is consistent with a Polycomb-associated chromatin state with potentially repressive function but does not establish direct mechanistic coupling between PRC1.1/H2AK119ub1 and H3K27me3 or a causal requirement of either feature for suppression of *AR* transcription. These findings identify a distinct PC-3 chromatin configuration in which absence of detectable FOXA1 occupancy at the distal module coexists with proximal PRC1.1/H2AK119ub1 enrichment and a broader TSS-centered H3K27me3-enriched state.

## 3. Discussion

The present findings indicate that the *AR* regulatory region is better considered not as a single promoter whose state is determined primarily by DNA methylation, but as a spatially heterogeneous system comprising functionally distinct distal and proximal segments. The distal region showed the greatest variability in DNA methylation and a high concentration of motifs for lineage-specific and signal-responsive TFs, whereas the region immediately surrounding the TSS remained predominantly hypomethylated but displayed marked differences in TF occupancy and chromatin state.

The proximal interval from −280 to +100 bp was particularly reproducible as a regulatory feature, concentrating H3K27ac, ERG-associated sites, and a proximal AR ChIP-seq signal in AR-positive models, whereas the same region in PC-3 cells was enriched in KDM2B, PCGF1, RNF2, and the repressive histone modification H2AK119ub1. Thus, prostate cancer progression appears to involve transitions among multiple context-dependent regulatory states rather than simple activation or silencing of the *AR* promoter.

### 3.1. Spatial Polarity of AR Promoter Methylation and Limitations of a Binary Model of Epigenetic Repression

Classical studies of AR-negative prostate cancer cell lines linked loss of AR expression to methylation of the promoter CpG island [10,11]. However, the present results indicate that this model is not universal. In primary tumors, the most pronounced methylation changes were concentrated distal to the TSS and consisted predominantly of reduced methylation, whereas the proximal TSS-associated region remained hypomethylated. Direct sample-level analysis in TCGA-PRAD further showed that associations between the 15 assayed promoter CpGs and AR mRNA were weak, and a joint methylation model had essentially no predictive performance in internal cross-validation. Thus, promoter methylation alone provides limited explanatory value for AR expression in primary prostate tumors.

More extensive distal demethylation was observed in the AR-negative neuroendocrine NCI-H660 model and across phenotypically distinct mCRPC states. In the WCDT cohort, all eight promoter-overlapping regional records showed lower methylation in the investigated subtype than in its contrast-specific ARPC reference. Because DMR boundaries and ARPC regional estimates were comparison-specific, and because the DNPC and SCNPC groups were small, these data do not establish an ordered methylation gradient among phenotypes. Instead, they support a recurrent direction of AR-promoter methylation remodeling across divergent lineage-plasticity states. A low methylation level alone is therefore not sufficient to indicate transcriptional activity of AR.

This pattern is consistent with current concepts of epigenetic reprogramming during prostate cancer progression. Genome-wide studies of mCRPC have demonstrated extensive remodeling of methylation across the *AR* locus and other regulatory regions [9]. Pomerantz et al. showed that metastatic progression is accompanied by broad reprogramming of the AR cistrome and H3K27ac landscape, with reactivation of regulatory programs related to prostate development [49]. A later study by Zhang et al. demonstrated increased use of alternative promoters during prostate cancer progression, with promoter activity associated with AR, FOXA1, and changes in DNA methylation [50]. Davies et al. further showed that the development of a therapy-resistant lineage-infidelity state is accompanied by substantial remodeling of chromatin architecture and the AR-dependent transcriptional program [51].

Importantly, DNA hypomethylation is a recognized feature of prostate cancer progression. Yegnasubramanian et al. showed that global DNA hypomethylation becomes particularly pronounced at the metastatic stage [52]. Accordingly, the distal demethylation observed in our analysis should be interpreted primarily as a manifestation of epigenetic remodeling, whose functional significance depends on the regulatory context and is shaped by the local chromatin architecture of the promoter region.

### 3.2. The Distal FOXA1 Module and Remodeling of Lineage-Specific Regulation

One of the most reproducible elements of the distal region was the FOXA1 module at −751 to −678 bp, containing ChIP-supported FOXA1 motifs at −751, −717, and −685 bp. Within-sample-normalized quantitative analysis showed higher FOXA1 signal in primary tumors than in matched normal tissue in both distal windows examined. The AR-associated FOXA1 signal also remained relatively enriched in mCRPC-derived PDXs after within-sample normalization; however, this cross-series comparison should not be interpreted as a direct measure of greater absolute FOXA1 occupancy because clinical tissues and PDXs differ in cellular composition and experimental context.

This finding is consistent with the results of Pomerantz et al., who showed that many regulatory elements that become active during metastatic progression are not formed de novo but are already occupied by pioneer factors such as FOXA1 and HOXB13 at earlier stages and are subsequently incorporated into a reprogrammed AR-dependent transcriptional network [49]. Additional evidence that chromatin remodeling can enhance chromatin accessibility and stabilize AR–FOXA1 interactions further emphasizes the dependence of FOXA1-associated regulation on co-regulatory and chromatin context [53].

In the 11 patients with paired measurements of both marks, the tumor-associated change in distal FOXA1 signal showed an exploratory positive association with proximal H3K27ac change, whereas the distal H3K27ac association was weaker. The difference between the proximal and distal correlations was not statistically established, and the result should therefore be interpreted as evidence of coordinated inter-individual remodeling rather than proof of spatially specific coupling.

Nevertheless, FOXA1 binding at the investigated site is not required for maintenance of every AR-positive state. C4-2B retains an AR-positive castration-resistant phenotype and strong proximal H3K27ac despite the absence of detectable FOXA1 occupancy within the −751 to −678 bp module. This is consistent with the previously described context dependence of FOXA1 effects on AR expression: FOXA1 depletion in some models can increase rather than decrease AR expression or the abundance of particular AR splice variants [18,19]. FOXA1 should therefore be considered an important component of specific *AR* regulatory configurations rather than a universal switch controlling its transcription.

Further transition toward AR-independent states may involve more extensive remodeling of the pioneer TF network. Wang et al. showed that in several AR-independent prostate cancer states, FOXA2 occupies regulatory elements distinct from FOXA1-dependent elements of luminal prostate cancer and cooperates with JUN/AP-1 in transcriptional reprogramming and lineage plasticity [54]. Loss of FOXA1 binding should therefore be interpreted as a potential component of a broader change in cellular identity rather than as an isolated mechanism of *AR* silencing.

### 3.3. The Proximal AR Promoter as a Center of Context-Dependent Regulatory Switching

In contrast to the distal segment, the most reproducible differences in *AR* promoter chromatin state were spatially concentrated immediately around the TSS. Of 145 H3K27ac-positive ChIP-seq datasets, 137 (94.5%) contained a peak overlapping the −280 to +100 bp interval. Proximal H3K27ac was particularly reproducible in the AR-positive VCaP and C4-2B models, whereas this active mark was rare or absent in the AR-negative PC-3 and DU145 models. The −280 to +100 bp region can therefore be considered the principal chromatin hub of the transcriptionally competent promoter state.

A particularly complex organization of this hub was observed in VCaP cells. ERG showed reproducible binding to the *AR* promoter: peaks were detected in 12/24 successfully analyzed datasets and overlapped the proximal motifs at −280, −225, −104, and −82 bp. In the same region, one of the most reproducible AR ChIP-seq signals spanned −308 to +266 bp and was detected in 19/123 VCaP datasets.

By contrast, the AREs selected by motif screening were located predominantly much farther upstream, at −1768, −1694, −1447, and −1091 bp. Thus, the strong proximal AR ChIP-seq signal did not coincide with the high-affinity AR motifs identified by JASPAR. Given the previously reported AR–ERG interaction [34] and binding of both factors within this locus, their spatial overlap is consistent with a shared proximal regulatory configuration, although direct AR–ERG interaction within this locus remains to be demonstrated.

An additional layer of plasticity may arise from overlap between motifs recognized by different ETS-family factors. Two predicted ETS1 motifs spatially coincide with ERG motifs at −280 and −225 bp, creating the potential for alternative occupancy of the same cis-regulatory sequences.

This possibility is particularly interesting in the context of functional data. In the study by Yu et al., ERG depletion increased AR mRNA and protein levels [34], whereas ETS1 depletion in another study reduced *AR* mRNA and increased methylation of its promoter region [35]. These effects are directionally opposite, although direct ETS1 binding to this specific promoter region was not examined in that study.

The proximal *AR* region may therefore be viewed not as a fixed ERG-binding element but as a potentially dynamic ETS-dependent regulatory module. In the ERG-positive VCaP context, overlapping sites may be preferentially occupied by ERG-containing complexes, whereas changes in the expression or activity of ETS factors could alter the composition of bound regulators. The proposed ERG→ETS1 switch remains a working hypothesis requiring direct experimental validation.

Overall, the H3K27ac-rich proximal region of *AR* is best considered a multifactorial regulatory hub whose state is determined by the combination of chromatin modifications and context-dependent TF occupancy.

### 3.4. PC-3 Combines a TSS-Centered H3K27me3-Enriched State with Proximal PRC1.1/H2AK119ub1

A notable observation is the coexistence in PC-3 cells of a broad TSS-centered H3K27me3-enriched configuration with pronounced proximal enrichment of KDM2B, PCGF1, RNF2, and H2AK119ub1 at the *AR* promoter.

PRC1.1 is a variant non-canonical PRC1 complex that can be recruited to unmethylated CpG-rich sequences through KDM2B [46]. Importantly, PRC1.1 occupancy is not intrinsically repressive and has also been reported at active genes [47]. In PC-3, the proximal PRC1.1/H2AK119ub1-associated configuration occurs within a broader H3K27me3-enriched chromatin context.

Within the proximal −280 to +100 bp interval, KDM2B, PCGF1, and RNF2 showed coordinated enrichment, whereas H2AK119ub1 displayed strong and nearly identical accumulation in two independent replicates. In contrast, no reproducible coordinated enrichment of these components was observed within the distal FOXA1 module at −751 to −678 bp. The PRC1.1/H2AK119ub1-associated configuration identified here is therefore preferentially localized around the TSS. This proximal PRC1.1/H2AK119ub1 focus therefore lies within a wider H3K27me3-enriched chromatin context.

PRC1 has previously been functionally linked to AR-negative double-negative prostate cancer. Using genetic and pharmacological approaches, Su et al. showed that PRC1 promotes the metastatic potential of DNPC by supporting stem-like properties and immune suppression [48]. Our data complement this observation by identifying accumulation of ncPRC1.1-associated components together with H2AK119ub1 within the proximal AR promoter in PC-3 cells.

The observed state can therefore be considered a locus-specific, multilayered Polycomb-associated configuration, the functional significance of which requires experimental validation. Importantly, the cross-sectional public datasets do not establish whether H3K27me3 deposition precedes, follows, or is mechanistically independent of PRC1.1/H2AK119ub1 accumulation.

### 3.5. Working Model of AR Promoter Remodeling During Progression and Lineage Plasticity

Taken together, the data support a working model in which the distal and proximal segments of the *AR* promoter undergo distinct but potentially coupled changes.

In primary adenocarcinoma, methylation loss was particularly pronounced within the region containing the distal FOXA1 module at −751 to −678 bp. At the same time, quantitative ChIP-seq analysis showed increased FOXA1 occupancy at this site relative to normal tissue, followed by further signal enrichment in mCRPC-derived PDXs. These spatially coincident changes are compatible with a model in which distal demethylation creates a more permissive epigenetic context for use of the FOXA1 module.

The correlation between changes in FOXA1 and H3K27ac was stronger for the proximal than for the distal region, but the difference between the two correlations was not statistically significant. In 31 matched normal–tumor pairs, H3K27ac did not show a statistically significant overall change in either the distal or proximal region. These findings therefore suggest possible coordination between FOXA1 and H3K27ac changes but do not establish a region-specific relationship or a causal mechanism.

With deeper lineage plasticity, distal demethylation may persist or become more extensive after loss of the AR-dependent transcriptional program. This is particularly evident in NCI-H660 and in AR-low/AR-negative mCRPC phenotypes. Distal demethylation therefore appears to represent a permissive feature of epigenetic plasticity, but is not sufficient for either FOXA1 binding or *AR* expression.

C4-2B further demonstrates that absence of detectable FOXA1 occupancy is itself insufficient to silence *AR*: this model retains an AR-positive phenotype and strong proximal H3K27ac.

In PC-3 cells, the proximal region adopts a distinct repressive configuration: a broad TSS-centered H3K27me3-enriched state coexists with local enrichment of KDM2B, PCGF1, RNF2, and H2AK119ub1. By contrast, DU145 is also AR-negative but lacks a comparable H3K27me3 profile. Transition toward AR independence therefore does not require a single uniform Polycomb state; different AR-negative models can reach transcriptionally silent or low-activity states through distinct chromatin configurations.

The C4-2B data further indicate that individual components of this remodeling can become uncoupled depending on cellular context.

### 3.6. Spatial and Epigenetic Polarity of the AR Promoter as an Integrative Model

Collectively, the present results support a model of spatial and epigenetic polarity of the *AR* promoter.

The distal segment is characterized primarily by DNA methylation plasticity and context-dependent use of lineage-specific and signal-responsive TFs. A prominent feature of this region is the FOXA1 module at −751 to −678 bp, which shows progression-associated enrichment of FOXA1 signal but is not required for maintenance of every AR-positive state. The proximal region, particularly the −280 to +100 bp interval, represents the principal regulatory hub in which TF and chromatin signals converge. In AR-positive states, this region is predominantly characterized by H3K27ac and may include an AR–ERG-containing regulatory configuration. In PC-3 cells, the same region lies within a broader H3K27me3-enriched domain and additionally adopts a sharply localized PRC1.1/H2AK119ub1-associated state with a potentially repressive function.

This spatial organization helps explain why individual epigenetic features do not have a uniform functional meaning. Distal demethylation can accompany either increased FOXA1 binding and AR-dependent regulation or an AR-negative state; loss of FOXA1 binding is compatible both with an active promoter in C4-2B and with AR-negative states; and loss of H3K27ac does not lead to a uniform H3K27me3 configuration. The strong H3K27me3 signal in PC-3, together with its absence or near-zero Input-corrected profile in DU145 and the analyzed mCRPC metastases, demonstrates that AR loss and H3K27me3 accumulation are not equivalent events.

Accordingly, the transcriptional state of *AR* appears to be determined not by a single epigenetic modification but by a context-dependent combination of spatially separated TF and chromatin modules.

### 3.7. Study Limitations

The principal limitations of this study arise from its integrative design. The public ChIP-seq, methylation, and other epigenomic datasets were generated in different studies and varied in experimental protocols, sequencing depth, data processing, and biological material. Frequencies of ChIP-seq peak detection across different cell models and clinical groups should therefore not be interpreted as direct quantitative comparisons of absolute occupancy.

The absence of longitudinal observations following the same cell population from an AR-positive to an AR-negative state prevents direct determination of the temporal sequence of the observed changes.

The WCDT analysis relies on published aggregated DSS results. For several regions, the reported nCG values could not be reconciled with the number of reference CpG dinucleotides, even when separate counting of the two strands was considered. In addition, ARPC methylation estimates differed between comparisons with identical regional boundaries. The audit results are provided in the reproducibility archive (supporting_tables/WCDT_DMR_records_8.csv; DOI: 10.5281/zenodo.22957702). Without the locus-level DSS input data, the reasons for these discrepancies remain unresolved. Accordingly, these values are interpreted as published estimates specific to each comparison and are not used to establish complete AR promoter demethylation or an ordered methylation gradient across phenotypes.

Co-localization of ChIP-seq peaks from different proteins does not by itself demonstrate their simultaneous presence within the same molecular complex. Likewise, coordinated enrichment of KDM2B, PCGF1, RNF2, and H2AK119ub1 in PC-3 is consistent with a PRC1.1/H2AK119ub1-associated chromatin configuration with potentially repressive function but does not establish its specificity for AR-negative cells or a causal role in suppression of AR transcription.

## 4. Materials and Methods

### 4.1. Study Design and Definition of the Analyzed AR Promoter Region

This study was designed as an integrative in silico analysis of the regulatory architecture of the AR promoter using publicly available genomic and epigenomic datasets. The analysis comprised prediction of potential TF-binding sites, assessment of DNA methylation, comparison of predicted motifs with TF ChIP-seq data, analysis of H3K27ac and H3K27me3 distribution, and a separate assessment of ncPRC1.1-associated components and H2AK119ub1 in PC-3 cells.

The primary region of interest was a 2501-bp interval extending from −2000 to +500 bp relative to the TSS of AR, corresponding to the 5′ end of RefSeq transcript NM_000044.6 (AR transcript variant 1). The TSS corresponds to chrX:66,763,863 in GRCh37/hg19 and chrX:67,544,021 in GRCh38/hg38; in GRCh38/hg38, the analyzed interval therefore corresponded to chrX:67,542,021–67,544,521. For cross-dataset spatial summaries, the promoter was operationally divided into a distal segment (−2000 to −281 bp) and a proximal segment (−280 to +500 bp). The −280-bp coordinate was used solely as a fixed reporting boundary for cross-dataset integration and was not interpreted as a statistically inferred methylation or motif transition. In TCGA-PRAD, the transition to the low-methylation TSS-proximal state was observed independently at approximately −300 bp, between the assayed CpGs at −342 and −259 bp.

Two additional functionally defined intervals were analyzed: the distal FOXA1 module at −751 to −678 bp and the proximal TSS-associated region at −280 to +100 bp.

### 4.2. Selection of TFs and JASPAR Motif Analysis

TFs were selected on the basis of published evidence of binding to regulatory regions of AR and/or functional effects on AR expression. Evidence from ChIP-based studies, functional CRISPR screens, gain- or loss-of-function experiments, and studies linking individual regulators to AR-dependent prostate cancer phenotypes was considered. Twenty-four TFs were included in the final motif analysis.

Potential TF-binding sites were screened within the −2000 to +500 bp sequence using position weight matrices from the JASPAR CORE vertebrate collection [55]. Both DNA strands were scanned. Nucleotide background frequencies were calculated directly from the analyzed promoter sequence. Position-specific scoring matrices were generated using a pseudocount of 0.8 and log-odds scores relative to the calculated nucleotide background; score distributions were calculated using 10,000 discrete levels.

Two significance levels were defined for motif classification: strict, *p* < 1 × 10^−4^; and supportive, 1 × 10^−4^ ≤ *p* < 5 × 10^−4^. Both strict and supportive matches were retained for the primary analysis, corresponding to an overall inclusion threshold of *p* < 5 × 10^−4^. The motif analysis was designed to map candidate TF-binding positions within the predefined AR promoter interval rather than to test motif over-representation or enrichment relative to a control sequence set.

When multiple overlapping matches for the same TF and DNA strand were present, they were merged into a single cluster. Thus, the reported cluster counts represent post-processed spatial predictions rather than the number of independent position-level threshold exceedances. The match with the lowest *p*-value was retained as the representative hit; when *p*-values were identical, the match with the higher PSSM score was selected. Genomic coordinates, position relative to the TSS, strand orientation, JASPAR matrix, and significance category were retained for each cluster. The final analysis contained 104 primary motif clusters, including 38 strict and 66 supportive clusters.

Clusters were assigned to the distal or proximal promoter segment according to the reported cluster start coordinate relative to the TSS. Clusters beginning at −280 bp were classified as proximal.

To assess spatial overlap between predicted motifs and CpG sequences, all CpG dinucleotides within the analyzed promoter interval were identified. A motif was considered to contain a CpG only when both nucleotides of the dinucleotide were entirely located within the motif interval. A total of 66 CpG dinucleotides were identified within the −2000 to +500 bp sequence.

### 4.3. DNA Methylation Analysis of the AR Promoter

DNA methylation of the AR promoter was evaluated in three independent datasets representing primary prostate adenocarcinoma, cellular models of AR-positive castration-resistant and AR-negative neuroendocrine disease, and phenotypically distinct states of mCRPC. Analyses were restricted to the −2000 to +500 bp promoter interval.

AR-status designations for the C4-2B and NCI-H660 cell models and the AR/NE phenotype assignments in the WCDT cohort were inherited from the corresponding source studies and were not independently remeasured or reclassified in the present analysis.

#### 4.3.1. TCGA-PRAD

Primary tumor-associated methylation changes were assessed using TCGA-PRAD HumanMethylation450 data [56] for 15 prespecified CpG sites within the −2000 to +500 bp AR promoter interval. The analyzed methylation matrix contained 498 Primary Tumor samples and 50 Solid Tissue Normal samples; cg25686125 had one missing tumor value (*n* = 497). Group-wise differences were summarized as mean β-value differences (Δβ = mean β(tumor) − mean β(normal)) and evaluated using Welch’s two-sample *t*-test with Benjamini–Hochberg correction across the 15 CpGs.

For the paired sensitivity analysis, 50 patients with both tumor and normal methylation data were identified. β-values were transformed to M-values as log_2_[β/(1−β)] after clipping β to the interval [10^−6^, 1−10^−6^]. Tumor–normal differences were tested using two-sided paired *t*-tests on M-values with Benjamini–Hochberg correction across the 15 CpGs. Mean paired β differences were reported as effect sizes, with 95% percentile confidence intervals estimated from 2000 patient-bootstrap resamples.

To evaluate the relationship between promoter methylation and AR expression at the sample level, TCGA-PRAD HumanMethylation450 and HiSeqV2 matrices were matched by TCGA patient identifier and tissue-type code, retaining at most one record per patient and tissue type in each assay. The methylation matrix contained 498 primary tumors and 50 normal samples, whereas the RNA matrix contained 497 tumors and 52 normal samples; their intersection comprised 497 primary tumors and 35 normal samples. AR RNA values were analyzed on the supplied log_2_(normalized count + 1) scale without an additional logarithmic transformation.

For each CpG, methylation β-value and AR RNA expression were associated using two-sided Spearman rank correlation separately in primary tumors and normal tissues, with Benjamini–Hochberg correction across the 15 CpGs within each tissue group. Percentile 95% confidence intervals for Spearman ρ were estimated from 2000 patient-bootstrap resamples. Separate single-predictor linear regression models were fitted to estimate the proportion of AR RNA variance explained by each CpG (*R*^2^). One tumor sample lacked cg25686125 methylation, resulting in *n* = 496 for that CpG.

To assess combined explanatory performance, an unadjusted multivariable linear model containing all 15 CpGs was fitted in 496 primary tumors with complete data. Training *R*^2^ and adjusted *R*^2^ were reported separately from predictive performance, which was evaluated using 20 repeats of five-fold cross-validation. Correlations between paired methylation and RNA changes were additionally evaluated in the 35 patients with both assays available in both tissue types, with Benjamini–Hochberg correction across the 15 CpGs.

#### 4.3.2. GSE158598: C4-2B and NCI-H660

To compare an AR-positive castration-resistant state with an AR-negative neuroendocrine state, processed normalized β-values from the Illumina Infinium MethylationEPIC dataset GSE158598 [57] were analyzed. The dataset comprised one C4-2B sample (GSM4803813) and one NCI-H660 sample (GSM4803814). Of the 15 CpG positions evaluated in TCGA-PRAD, 13 were represented on the EPIC array.

For each available CpG site, β-values were directly compared between C4-2B and NCI-H660. Because only one sample was available for each cell model, no formal statistical test was performed; this comparison was used as a descriptive assessment of the spatial methylation profile.

#### 4.3.3. WCDT mCRPC Cohort

To evaluate AR promoter methylation across phenotypically distinct mCRPC states, published WGBS/DSS results from the WCDT cohort [8] were analyzed. Samples were classified in the source study as ARPC (AR+/NE−; *n* = 40), ARLPC (AR-low/NE−; *n* = 21), AMPC (AR+/NE+; *n* = 4), DNPC (AR−/NE−; *n* = 2), and SCNPC/NEPC (AR−/NE+; *n* = 3). The published subtype assignments were used directly and were not recalculated in the present study.

The released aggregate DSS object (DMRs.data in DMR.data.RData) was queried for records overlapping chrX:67,542,021–67,544,521 (GRCh38/hg38), corresponding to −2000 to +500 bp relative to the AR TSS at chrX:67,544,021. Source DMR boundaries and regional DSS statistics were retained without recalculation. Region width was calculated as end − start + 1, and the number of unique reference CpG dinucleotides contained within each full source interval was determined independently from the GRCh38 reference sequence.

For each extracted record, the published DSS regional methylation estimates were reproduced as reported, with meanMethy1 corresponding to the investigated subtype, meanMethy2 to the ARPC reference, and diff.Methy to subtype minus ARPC. The source areaStat value was retained as the DSS regional summary statistic, representing the sum of locus-level test statistics across the reported region; areaStat was not treated as a regional *p* value or FDR.

HM450 and EPIC CpG positions were mapped to GRCh38 and intersected with the full source DMR intervals to assess spatial correspondence with the array-based analyses. Each subtype-versus-ARPC contrast was treated independently because the released regional boundaries and ARPC estimates were comparison-specific. Individual sample-level WGBS methylation values were not available in the released aggregate object; consequently, no additional patient-level statistical testing or formal test of methylation ordering across phenotypic states was performed.

#### 4.3.4. AR-Status Classification and Source-Study Definitions

AR status of the cell-line models and molecular subtypes of the WCDT cohort was adopted from the corresponding source studies and was not independently reassessed in the present study. The mCRPC phenotypic framework was originally established by Labrecque et al. [44], who classified tumors according to RNA-seq profiles of AR-associated and neuroendocrine transcriptional programs. In that study, ARPC and ARLPC were distinguished quantitatively on the basis of AR and KLK3 expression, with ARLPC defined by an AR log_2_(FPKM) value < 4.0 or a KLK3 log_2_(FPKM) value < 2.0.

For the WCDT cohort analyzed here, these expression thresholds were not reapplied. Instead, the published molecular-subtype assignments reported by Lundberg et al. [8] were used directly. In the source study, WCDT tumors were classified by hierarchical clustering of RNA-seq profiles using previously defined AR-associated, neuroendocrine, and squamous-differentiation gene panels, resulting in five transcriptional states: AR+/NE−, AR+/NE+, AR-low/NE−, AR−/NE+, and AR−/NE−, corresponding here to ARPC, AMPC, ARLPC, SCNPC/NEPC, and DNPC, respectively.

For cell-line comparisons, AR-positive or AR-negative designations were likewise retained as reported in the corresponding source publications; tumor-cohort expression thresholds were not applied to cell models. Accordingly, AR-status labels in the cell-line and WCDT analyses represent previously established molecular or phenotypic classifications rather than measurements generated in the present study. This differs from the TCGA-PRAD analysis, in which promoter methylation was directly related to measured AR RNA expression in sample-matched specimens.

### 4.4. ChIP-Seq Analysis of TF Binding

Standardized hg38 ChIP-seq peak calls from ChIP-Atlas [58] were used to assess experimental binding of selected TFs within the AR promoter. Analyses were restricted to chrX:67,542,021–67,544,521, corresponding to −2000 to +500 bp relative to the TSS. ChIP-Atlas peaks were queried using a threshold of *q* < 1 × 10^−5^.

For each ChIP-seq dataset, the presence of at least one peak overlapping the promoter interval was determined programmatically. For the corresponding TF, promoter peaks were then tested for overlap with the motifs retained from the JASPAR screening. For motif–ChIP integration, the primary analysis was restricted to strict-tier JASPAR motifs (*p* < 1 × 10^−4^). A motif was considered ChIP-overlapping when an AR-promoter ChIP-seq peak for the same TF intersected the corresponding motif interval. Supportive-tier motifs (1 × 10^−4^ ≤ *p* < 5 × 10^−4^) were retained as predicted sites but were not included in the primary strict-tier motif–ChIP analysis.

To determine whether the observed motif–ChIP overlap exceeded that expected from the local promoter architecture, a per-TF circular permutation analysis was performed. For each TF, the complete set of strict-tier motif intervals was circularly shifted within the 2501-bp AR promoter while preserving motif number, width, and relative spacing, whereas the observed ChIP-seq peak intervals were kept fixed. For each of 10,000 permutations, the number of successfully queried datasets containing at least one promoter peak overlapping at least one shifted strict-tier motif was recorded. Empirical *p*-values were calculated as (1 + number of permutations with a statistic at least as large as observed)/(10,001).

A total of 1121 successfully queried ChIP-seq datasets representing 10 TFs were included in the final analysis. Datasets returning query errors were excluded from the denominator and were not classified as ChIP-seq-negative. Two E2F1 datasets and eight ERG datasets returned query errors. No eligible TFAP2C ChIP-seq datasets were available in the analyzed collection.

When several closely spaced motifs were overlapped by the same broad ChIP-seq peak, site-specific counts were not summed. In particular, the FOXA1 motifs at −751, −717, and −685 bp were considered a compact distal module because they were predominantly overlapped by the same broad ChIP-seq peaks in positive datasets.

Promoter ChIP-seq signals that did not overlap the selected JASPAR motifs were analyzed separately. Overlapping peak intervals for the same TF within a biological model were merged into representative regions, and the number of datasets supporting each merged region was determined. This procedure was used, among other analyses, to characterize the proximal AR ChIP-seq signal that did not overlap the selected distal androgen response element motifs.

### 4.5. Quantitative Analysis of FOXA1 and H3K27ac in Clinical Samples and mCRPC-Derived PDXs

The distal FOXA1 module was further examined using quantitative ChIP-seq data from GSE130408 [49,59], including normal prostate tissue, primary prostate tumors, and mCRPC-derived LuCaP patient-derived xenografts (PDXs). The FOXA1 analysis included 14 normal prostate samples, 13 primary tumors, and 15 mCRPC-derived PDXs. Thirteen patient-matched normal-primary tumor pairs were available, comprising five pairs from the Dana-Farber Cancer Institute (DF) series and eight pairs from the Netherlands Cancer Institute (NKI) series.

Quantitative H3K27ac analysis was performed in a separate set of 31 matched normal-primary tumor pairs, comprising 24 DF and seven NKI pairs. Eleven patients (four DF and seven NKI) had paired FOXA1 and H3K27ac measurements available and were included in the integrated association analysis.

Processed BigWig tracks from GSE130408 were analyzed in their native GRCh37/hg19 coordinate system. For this dataset, the AR TSS was defined at chrX:66,763,863. The distal FOXA1 interval at −751 to −678 bp corresponded to chrX:66,763,112-66,763,185, whereas the proximal interval at −280 to +100 bp corresponded to chrX:66,763,583-66,763,963. For integration with datasets analyzed in GRCh38/hg38, regulatory features were aligned according to their position relative to the corresponding AR TSS in each genome assembly rather than by directly combining genomic coordinates from different assemblies.

Regional signal was calculated as a length-weighted mean across each specified interval. The corresponding regional signal integral was obtained by summing the product of signal intensity and the length of overlap for all BigWig segments intersecting the interval.

FOXA1 signal was evaluated in the two distal intervals examined in the original analysis: a 201-bp window spanning −814 to −614 bp and a narrower 74-bp window spanning the −751/−717/−685 bp FOXA1 motif cluster (−751 to −678 bp). For cross-series comparisons, the regional FOXA1 signal integral was additionally normalized within each sample to the total FOXA1 signal across canonical hg19 chromosomes 1-22 and X and expressed as parts per million (ppm) of total FOXA1 signal. Paired normal-primary tumor differences were evaluated using two-sided Wilcoxon signed-rank tests, whereas comparisons between clinical tissues and mCRPC-derived PDXs were performed using two-sided Mann–Whitney U tests. Mean paired FOXA1 changes and their 95% confidence intervals were estimated by bootstrap resampling.

To compare FOXA1 signal with promoter chromatin state, H3K27ac signal was quantified in two intervals: the distal −751 to −678 bp window and the proximal −280 to +100 bp window. For each matched normal-tumor pair, the tumor-associated change was calculated as:

Δsignal = signal(tumor) − signal(normal).

Paired normal-tumor differences in H3K27ac were evaluated using two-sided Wilcoxon signed-rank tests. Mean paired changes and their 95% confidence intervals were estimated by bootstrap resampling. Among the 11 patients with paired measurements of both FOXA1 and H3K27ac, associations between the tumor-associated change in distal FOXA1 signal and changes in H3K27ac within the distal and proximal intervals were evaluated using Spearman rank correlation. Because of the small sample size, statistical significance of the Spearman correlations was additionally assessed using two-sided permutation testing, and 95% confidence intervals for the correlation coefficients were estimated by bootstrap resampling.

Because the FOXA1-distal H3K27ac and FOXA1-proximal H3K27ac correlations were calculated in the same patients and shared the FOXA1 variable, their difference was evaluated as a comparison of dependent overlapping correlations. The correlation between distal and proximal H3K27ac changes was included in an approximate Williams-type test applied to rank-transformed variables. The difference between the two Spearman correlation coefficients was additionally evaluated by bootstrap resampling.

Robustness of the FOXA1-proximal H3K27ac association was assessed by leave-one-out sensitivity analysis, in which each patient was sequentially excluded and the Spearman correlation coefficient was recalculated. Permutation and bootstrap procedures used 100,000 resamples.

### 4.6. Analysis of H3K27ac and H3K27me3

The H3K27ac state of the AR promoter was screened using standardized ChIP-seq peak calls obtained from ChIP-Atlas [58]. The promoter interval was hg38 chrX:67,542,021–67,544,521 (−2000 to +500 bp relative to the TSS), and a ChIP-Atlas threshold of *q* < 1 × 10^−5^ was used. A total of 516 H3K27ac datasets were surveyed. H3K27ac-positive datasets were additionally tested for overlap with the proximal −280 to +100 bp interval.

H3K27me3 was analyzed quantitatively using 31 matched H3K27me3 ChIP and Input pairs with technically valid Input tracks. Candidate datasets were restricted to unperturbed biological states relevant to the progression analysis. Pairs were excluded if the matched Input track was identically zero across the analyzed AR region. ChIP-Atlas BigWig signals were used in their RPM-normalized form. Profiles were extracted in 100-bp bins across ±10 kb relative to the hg38 *AR* TSS at chrX:67,544,021. For each bin, the primary Input-corrected signal was calculated as ΔH3K27me3 = RPM_ChIP_ − RPM_Input_. The resulting profiles were smoothed with a centered 500-bp window. Subtraction of normalized Input from normalized ChIP signal is an established ChIP-seq representation and avoids the instability of fold ratios when Input approaches zero [60,61,62]. No pseudocount was added.

For group-level visualization, a study-first aggregation strategy was used: replicate/sample profiles were summarized by the median within each study block and then by the median across study blocks, preventing studies with more samples from dominating the group profile. Window-level ΔRPM values were calculated for the −2000/+500 bp promoter interval and for TSS-centered ±2, ±5, and ±10 kb windows. Primary normal prostate tissue, PrEC, primary PCa GS6 and GS8, LNCaP AR-dependent, LNCaP androgen-independent/CRPC, DU145, PC-3, and mCRPC metastases were retained as distinct biological groups. PrEC was treated as a primary cultured epithelial reference rather than as equivalent to intact normal tissue.

RWPE-1 from GSE63094 was analyzed separately as an independent benign immortalized epithelial reference because it was generated in a different genome assembly and was not part of the 31-pair study-first set. The dataset comprised two H3K27me3 ChIP replicates and one matched Input and was analyzed in its native GRCh37/hg19 coordinates using the *AR* TSS at chrX:66,763,863; all results were expressed relative to the TSS for spatial comparison. The mean of the two H3K27me3 replicates was used before Input subtraction. Quantitative ΔRPM values were interpreted descriptively across independent studies because RPM normalization and matched-Input subtraction do not remove all differences in antibody performance, sequencing depth distribution, chromatin preparation, and other batch effects; no spike-in calibration was available.

Accordingly, cross-study conclusions were based primarily on the spatial shape, sign, and reproducibility of Input-corrected profiles across independent study blocks rather than on formal statistical testing of absolute ΔRPM magnitude. Quantitative ΔRPM profiles and author-provided broad intervals, where available, were treated as complementary analytical layers.

### 4.7. Reprocessing of ncPRC1.1-Associated and H2AK119ub1 ChIP-Seq Data in PC-3 Cells

Public PC-3 ChIP-seq data for KDM2B, PCGF1, RNF2, and two H2AK119ub1 libraries were obtained from GSE131829 [48] and reprocessed from FASTQ files. The Input library GSM3820351 was used as a shared control for all five ChIP libraries. For the paired-end RNF2 and Input runs, only read 1 (R1) was used, and all libraries were processed as single-end data. Downloaded FASTQ files were verified against the checksums recorded in the sample manifest. Reads were aligned to the complete UCSC hg38 reference using Rsubread 2.26.0 in DNA mode with unique alignment reporting (unique = TRUE; nBestLocations = 1). No separate adapter or quality trimming was applied. Primary mapped alignments passing vendor quality control were retained at MAPQ ≥ 20, excluding MAPQ = 255; secondary and supplementary alignments were excluded. Exact-coordinate duplicates were collapsed by reference sequence, strand, and aligned 5′ coordinate. Library depth was defined as the number of retained, deduplicated alignments across all reference sequences, including chrM and alternate or unplaced sequences.

The analysis used the AR TSS at chrX:67,544,021 (hg38; 1-based). The distal window extended from −751 to −678 bp relative to the TSS (chrX:67,543,270–67,543,343; 74 bp), and the proximal window extended from −280 to +100 bp (chrX:67,543,741–67,544,121; 381 bp). Both interval endpoints were included. Alignments were extended from their aligned 5′ positions in the strand-specific direction to a prespecified length of 120 bp, and each extended alignment overlapping a window was counted once.

Depth-normalized enrichment relative to Input was calculated separately for each ChIP library and window as:

Enrichment = (*N*_ChIP,region_/*N*_ChIP,total_)/(*N*_Input,region_/*N*_Input,total_).

Here, *N*_region_ denotes the count of extended alignments overlapping the window, and *N*_total_ denotes the corresponding genome-wide count after the same filtering and deduplication. No pseudocount was added; a zero or invalid Input denominator was reported as unavailable. The primary estimates in Table 10 used the 120-bp extension. Extension lengths of 150, 180, and 200 bp were examined separately as sensitivity analyses and were not treated as biological replicates or pooled into the primary estimate. The 120-bp setting was prespecified and was not estimated by a new cross-correlation analysis in this reanalysis. Enrichment ratios were interpreted descriptively because local counts were low and all ChIP libraries shared one Input control; co-enrichment does not establish physical co-complexation or a causal role in AR repression.

### 4.8. Statistical Analysis and Cross-Dataset Integration

Statistical procedures were selected according to the structure of each dataset. TCGA-PRAD group-wise CpG comparisons were evaluated using Welch’s two-sample *t*-tests on β-values, while paired tumor–normal sensitivity analyses used two-sided paired *t*-tests on M-values; Benjamini–Hochberg correction was applied across the 15 predefined promoter CpGs. Methylation–AR RNA associations were assessed using two-sided Spearman rank correlation with Benjamini–Hochberg correction and bootstrap confidence intervals. Paired clinical FOXA1 and H3K27ac comparisons were evaluated using two-sided Wilcoxon signed-rank tests, and independent comparisons involving PDXs used two-sided Mann–Whitney U tests. Unless otherwise specified, *p* < 0.05 was considered statistically significant.

No formal statistical comparison was performed for GSE158598 because only one C4-2B and one NCI-H660 sample were available. Additional DMR-level testing was not performed for the WCDT cohort because individual sample-level methylation values were unavailable in the analyzed regional summary data.

Cross-dataset integration was performed using coordinates relative to the AR TSS. For datasets represented in different genome assemblies, regulatory features were aligned by their position relative to the corresponding TSS in each assembly, enabling spatial comparison of motif, ChIP-seq, DNA methylation, and histone-modification signals. The principal integrative intervals were the distal FOXA1 module at −751 to −678 bp and the proximal regulatory interval at −280 to +100 bp.

### 4.9. Software and Computational Environment

Statistical analyses, data processing, and visualization were performed in R version 4.6.1 (R Foundation for Statistical Computing, Vienna, Austria) using RStudio Desktop version 2026.09.0+174 (Posit Software, PBC, Boston, MA, USA) and Python version 3.14.8 (Python Software Foundation, Beaverton, OR, USA). Custom scripts were used for data processing, statistical analysis, genomic interval handling, cross-dataset integration, and visualization. The web resources used were JASPAR (https://jaspar.elixir.no/), ChIP-Atlas (https://chip-atlas.org/), and the UCSC Genome Browser (https://genome.ucsc.edu/); the Rsubread alignment package version is specified in Section 4.7.

## 5. Conclusions

The present study expands the conventional view of epigenetic regulation of AR. Our findings indicate that changes in AR activity during prostate cancer progression are not determined by a single epigenetic event, but rather by remodeling of spatially separated regulatory modules involving DNA methylation, binding of lineage-specific TFs, and context-dependent changes in the chromatin state of the proximal promoter. This model provides a framework that integrates enhanced AR-dependent regulation during the development of mCRPC with the subsequent loss of AR signaling during lineage plasticity within a unified, but context-dependent, promoter architecture.

## Figures and Tables

**Table 1:** Transcriptional and architectural factors associated with the regulation of AR expression and activity.

Factor	Type of Evidence	Summary of Role in AR Expression	References
SP1	ChIP/GC-box binding analysis; mutational and functional promoter analysis	Supports basal AR transcription and influences TSS selection.	[13,14]
FOXA1	Immunohistochemical correlation; knockdown in LNCaP and 22Rv1; mRNA and protein analyses	The association with AR expression is context-dependent: FOXA1 and AR levels are positively correlated in tumors, whereas FOXA1 knockdown may increase AR or specific AR variants.	[17,18,19]
GATA2	ChIP; RNAi; mRNA and protein analyses before and after androgen stimulation	Positive regulator supporting basal and hormone-induced AR expression.	[20]
HOXB13	Functional CRISPR-Cas9 screening; knockdown; mRNA and protein analyses	Required to maintain AR expression in AR-positive PCa models.	[21]
TFAP2C	Functional CRISPR-Cas9 screening; knockdown; mRNA and protein analyses	Positively associated with maintenance of AR expression in AR-positive cell models.	[21]
NKX3-1	Tissue correlation; ChIP; knockdown with two siRNAs; mRNA and protein analyses	Forms a positive regulatory loop with AR; binds an intragenic region approximately +79 kb from the TSS and supports AR expression.	[22,23]
AR	ChIP; mutational analysis of AREs; histone modification and transcription analyses	Ligand-activated AR participates in negative autoregulation of its own transcription through elements in the 5′ UTR and second intron.	[24,25]
NR3C1 (GR)	Enzalutamide-resistance models; dexamethasone activation; genetic and pharmacological inhibition	Functionally substitutes for AR at a subset of target genes and supports antiandrogen resistance; a direct effect on AR transcription has not been established.	[26]
CTCF	in situ Hi-C; analysis of Pol II-associated chromatin contacts; ChIP	Architectural regulator of AR-locus organization; a possible association with locus amplification and generation of truncated transcripts has not been functionally demonstrated.	[27,28]
E2F1	Overexpression; mRNA and protein analyses; promoter reporter assay; ChIP; analysis of the E2F1–DNMT1 axis	Negatively regulates AR transcription, likely as part of a repressive complex with pocket proteins and DNMT1.	[29,30]
MYC	Knockdown; analysis of pre-mRNA and mature transcripts; promoter reporter assays	Positively regulates expression of full-length AR and its splice variants.	[31]
KLF4	ChIP-qPCR; DHT/antiandrogen modulation; knockdown; mRNA and protein analyses	Positive, context-dependent regulator of AR; DHT-dependent binding was detected at −347 and +647 bp.	[32]
KLF5	Knockdown; mRNA and protein analyses; promoter reporter assay; ChIP	Directly activates AR transcription; binding was confirmed within the proximal region at −107 to −93 bp.	[33]
ERG	ChIP-seq and ChIP-PCR; overexpression and knockdown; mRNA and protein analyses; promoter reporter assay	Negative regulator of AR; binds the promoter region and represses AR transcription.	[34]
ETS1	Knockdown; mRNA analysis; assessment of promoter CpG-island methylation	Evidence is limited: ETS1 knockdown reduced AR mRNA and was accompanied by increased promoter methylation; direct binding was not investigated.	[35]
JUN (c-Jun)	Overexpression; promoter reporter assay; analysis of mature and nascent RNA and protein	Suppresses AR transcription and expression; direct promoter occupancy was not demonstrated in the studies considered.	[36,37]
STAT1	Expression analysis; TP53/RB1-deficient and therapy-resistant prostate cancer models; pharmacological inhibition of JAK/STAT1; stemness assays	Increased STAT1 expression is associated with reduced AR levels and acquisition of stem-like and neuroendocrine features, supporting its involvement in progression toward AR-low/AR-independent, therapy-resistant states; direct binding to the AR promoter was not demonstrated.	[38]
STAT5	Knockdown and constitutive activation; analysis of nascent pre-mRNA, AR-FL, and AR variants	Positively regulates transcription of AR and its splice variants in several PCa models.	[39]
RELA/p65–NFKB1/p50	Overexpression; promoter reporter assay; mRNA and protein analyses; NF-κB inhibition; ChIP-PCR	The canonical NF-κB complex can activate AR transcription; p50-containing complexes bind the 5′ regulatory region.	[40]
NFKB2/p52	Functional activation; analysis of AR activity and AR-FL/AR-variant expression	Enhances ligand-independent AR activity and expression of AR variants, including AR-V7, without substantially increasing full-length AR.	[41]
HIF1A	Immunohistochemical correlation in 149 clinical samples	HIF1A protein levels are positively associated with AR levels; a direct effect on AR transcription has not been established.	[42]
HIF2A	Immunohistochemical correlation in 149 clinical samples	HIF2A protein levels are positively associated with AR levels; the association was stronger than that observed for HIF1A.	[42]
TP53 (p53)	Activation and overexpression; siRNA; mRNA and protein analyses; ChIP-PCR after etoposide treatment	Negatively regulates AR expression. p53 association with the 5′ regulatory region was detected under genotoxic stress but not under basal conditions.	[43]

Abbreviations: ARE, androgen response element; AR-FL, full-length AR; AR-V7, AR splice variant 7; ChIP, chromatin immunoprecipitation; ChIP-seq, ChIP followed by sequencing; CRISPR, clustered regularly interspaced short palindromic repeats; DHT, dihydrotestosterone; GC, guanine–cytosine; Hi-C, high-throughput chromosome conformation capture; mRNA, messenger RNA; PCa, prostate cancer; PCR, polymerase chain reaction; Pol II, RNA polymerase II; qPCR, quantitative PCR; RNAi, RNA interference; siRNA, small interfering RNA; TSS, transcription start site; UTR, untranslated region.

**Table 2:** Results of JASPAR motif screening for 24 TFs within the AR promoter region.

TF	JASPAR Profile	Total Clusters, Strict/Supportive	Distal/Proximal	Best Nominal *p*
SP1	MA0079.5	13, 7/6	2/11	2.9 × 10^−6^
FOXA1	MA0148.5	5, 1/4	5/0	5.5 × 10^−5^
GATA2	MA0036.4	2, 1/1	2/0	7.3 × 10^−5^
HOXB13	MA0901.3	4, 2/2	4/0	3.5 × 10^−5^
TFAP2C	MA0524.3, MA0814.3, MA0815.1	10, 1/9	4/6	2.0 × 10^−5^
NKX3-1	MA0124.3	1, 0/1	1/0	2.7 × 10^−4^
AR	MA0007.2	4, 1/3	4/0	5.9 × 10^−5^
NR3C1	MA0113.2	6, 1/5	6/0	8.4 × 10^−5^
CTCF	MA0139.2, MA1929.2, MA1930.2	4, 0/4	1/3	2.0 × 10^−4^
E2F1	MA0024.3	4, 3/1	2/2	3.2 × 10^−5^
MYC	MA0147.4	0	0/0	—
KLF4	MA0039.5	11, 8/3	1/10	1.8 × 10^−5^
KLF5	MA0599.1	9, 6/3	1/8	2.0 × 10^−5^
ERG	MA0474.4	8, 2/6	2/6	2.5 × 10^−5^
ETS1	MA0098.4	4, 0/4	2/2	2.7 × 10^−4^
JUN	MA0488.2, MA0489.3	1, 0/1	1/0	3.5 × 10^−4^
STAT1	MA0137.4	4, 2/2	4/0	4.0 × 10^−5^
STAT5	MA0519.2, MA1624.2, MA1625.2	9, 3/6	9/0	5.2 × 10^−5^
RELA	MA0107.1	3, 0/3	1/2	1.0 × 10^−4^
NFKB1	MA0105.4	0	0/0	—
NFKB2	MA0778.2	1, 0/1	0/1	4.96 × 10^−4^
HIF1A	MA1106.2	0	0/0	—
HIF2A/EPAS1	MA2325.1	0	0/0	—
TP53	MA0106.3	1, 0/1	1/0	3.8 × 10^−4^

Note: Strict matches, *p* < 10^−4^; supportive matches, 10^−4^ ≤ *p* < 5 × 10^−4^. For spatial classification, an operational boundary at −280 bp relative to the TSS was used: distal segment, −2000 to −281 bp; proximal segment, −280 to +500 bp. Clusters were assigned according to the reported cluster start coordinate relative to the TSS; clusters starting at −280 bp were classified as proximal. Abbreviations: TF, transcription factor; TSS, transcription start site; bp, base pairs. JASPAR is the name of the TF-binding profile database; MA identifiers are JASPAR matrix accessions.

**Table 4:** Source-reported WCDT regional records overlapping the AR promoter.

Subtype	hg38 Interval (chrX)	Relative Interval (bp)	Width (bp)	Unique Reference CpGs (*n*)	AreaStat
ARLPC	67,543,271–67,543,426	−750 to −595	156	2	−54.925
AMPC	67,543,271–67,543,426	−750 to −595	156	2	−65.403
AMPC	67,544,285–67,544,581	+264 to +560	297	16	−176.087
DNPC	67,543,271–67,543,426	−750 to −595	156	2	−75.823
DNPC	67,543,843–67,544,528	−178 to +507	686	50	−537.801
SCNPC	67,543,271–67,543,535	−750 to −486	265	5	−102.809
SCNPC	67,543,762–67,544,120	−259 to +99	359	21	−222.709
SCNPC	67,544,210–67,544,524	+189 to +503	315	22	−221.531

Notes: Coordinates are 1-based GRCh38/hg38. Relative positions are reported with respect to the AR TSS. Unique reference CpGs represent CpG dinucleotides contained within each full source interval. areaStat values were reproduced from the published DSS output and were not recalculated; areaStat is a regional DSS summary statistic and is not a regional *p* value or FDR. Abbreviations: AMPC, amphicrine prostate cancer; ARLPC, AR-low prostate cancer; CpG, cytosine–phosphate–guanine dinucleotide; DNPC, double-negative prostate cancer; DSS, Dispersion Shrinkage for Sequencing data; FDR, false discovery rate; SCNPC, small-cell/neuroendocrine prostate cancer; TSS, transcription start site; WCDT, West Coast Dream Team; bp, base pairs.

**Table 5:** ChIP-seq occupancy within the AR promoter and overlap with strict- and supportive-tier JASPAR motifs in prostate-derived cell models, primary tissue, and mCRPC metastatic tissues (ChIP-Atlas *q* < 1 × 10^−5^).

TF	Biological Model/Tissue	Datasets, *N*	Promoter Peak, *n*/*N* (%)	Strict-Tier JASPAR Motif(s) with Same-TF ChIP-Seq Overlap	Supportive-Tier JASPAR Motif(s) with Same-TF ChIP-Seq Overlap (Descriptive)
CTCF	Prostate cancer cell line—22Rv1	12	4/12 (33.3%)	—	MA1929.2: −867 bp; 4/12 (33.3%)
CTCF	Prostate cancer cell line—C4-2	2	0/2	—	—
CTCF	Prostate cancer cell line—DU145	1	0/1	—	—
CTCF	Prostate cancer cell line—LNCaP	25	0/25 (0.0%)	—	—
CTCF	Prostate cancer cell line—PC-3	3	0/3	—	—
CTCF	Prostate cancer cell line—VCaP	8	1/8 (12.5%)	—	MA1929.2: −867 bp; 1/8 (12.5%)
CTCF	Non-malignant prostate cell line—RWPE-1	2	0/2	—	—
CTCF	Non-malignant prostate cell line—RWPE-2	2	0/2	—	—
CTCF	Primary prostate epithelial cells (PrEC)	3	0/3	—	—
CTCF	Primary prostate tissue	6	0/6 (0.0%)	—	—
CTCF	mCRPC metastasis—liver	1	0/1	—	—
CTCF	mCRPC metastasis—lymph node	2	0/2	—	—
FOXA1	Prostate cancer cell line—22Rv1	77	25/77 (32.5%)	MA0148.5: −685 bp; 25/77 (32.5%)	MA0148.5: −751, −717 bp; 25/77 (32.5%)
FOXA1	Prostate cancer cell line—C4-2	8	1/8 (12.5%)	MA0148.5: −685 bp; 1/8 (12.5%)	MA0148.5: −751, −717 bp; 1/8 (12.5%)
FOXA1	Prostate cancer cell line—C4-2B	6	0/6 (0.0%)	—	—
FOXA1	Prostate cancer cell line—DU145	5	0/5 (0.0%)	—	—
FOXA1	Prostate cancer cell line—LNCaP	123	31/123 (25.2%)	MA0148.5: −685 bp; 31/123 (25.2%)	MA0148.5: −751, −717 bp; 31/123 (25.2%)
FOXA1	Prostate cancer cell line—VCaP	49	33/49 (67.3%)	MA0148.5: −685 bp; 31/49 (63.3%)	MA0148.5: −751, −717 bp; 31/49 (63.3%)
FOXA1	mCRPC metastasis—liver	1	0/1	—	—
FOXA1	mCRPC metastasis—lung	1	1/1	—	—
FOXA1	mCRPC metastasis—lymph node	2	1/2	MA0148.5: −685 bp; 1/2	MA0148.5: −751, −717 bp; 1/2
GATA2	Prostate cancer cell line—LNCaP	19	0/19 (0.0%)	—	—
GATA2	Prostate cancer cell line—VCaP	4	0/4	—	—
HOXB13	Prostate cancer cell line—22Rv1	5	0/5 (0.0%)	—	—
HOXB13	Prostate cancer cell line—C4-2B	5	0/5 (0.0%)	—	—
HOXB13	Prostate cancer cell line—LNCaP	23	0/23 (0.0%)	—	—
HOXB13	Prostate cancer cell line—PC-3	1	0/1	—	—
HOXB13	Prostate cancer cell line—VCaP	12	1/12 (8.3%)	—	—
AR	Prostate cancer cell line—22Rv1	78	0/78 (0.0%)	—	—
AR	Prostate cancer cell line—C4-2	38	0/38 (0.0%)	—	—
AR	Prostate cancer cell line—C4-2B	2	0/2	—	—
AR	Prostate cancer cell line—DU145	11	0/11 (0.0%)	—	—
AR	Prostate cancer cell line—LNCaP	323	8/323 (2.5%)	—	—
AR	Prostate cancer cell line—PC-3	7	0/7 (0.0%)	—	—
AR	Prostate cancer cell line—VCaP	123	19/123 (15.4%)	—	—
AR	mCRPC metastasis—liver	1	1/1	—	—
AR	mCRPC metastasis—lung	1	1/1	—	—
AR	mCRPC metastasis—lymph node	2	2/2	—	—
NR3C1/GR	Prostate cancer cell line—22Rv1	28	0/28 (0.0%)	—	—
NR3C1/GR	Prostate cancer cell line—LNCaP	5	0/5 (0.0%)	—	—
NR3C1/GR	Prostate cancer cell line—VCaP	26	1/26 (3.8%)	—	—
TP53	Prostate cancer cell line—22Rv1	2	0/2	—	—
TP53	Prostate cancer cell line—LNCaP	12	0/12 (0.0%)	—	—
TP53	Prostate cancer cell line—VCaP	4	0/4	—	—
E2F1	Prostate cancer cell line—LNCaP	11	4/11 (36.4%)	MA0024.3: −264 bp; 2/11 (18.2%)	—
ERG	Prostate cancer cell line—VCaP	24	12/24 (50.0%)	—	MA0474.4: −280, −225, −104, −82, +222, +264 bp; Any motif: 12/24 (50.0%); −280: 6/24 (25.0%); −225: 10/24 (41.7%); −104: 12/24 (50.0%); −82: 12/24 (50.0%); +222: 1/24 (4.2%); +264: 1/24 (4.2%)
ERG	Non-malignant prostate cell line—RWPE-1	9	0/9 (0.0%)	—	—
KLF5	Prostate cancer cell line—R1-AD1	4	0/4	—	—
KLF5	Prostate cancer cell line—R1-D567	2	0/2	—	—

Notes: A total of 1121 successfully queried hg38 ChIP-seq datasets were analyzed for 10 TFs. AR promoter interval: hg38 chrX:67,542,021–67,544,521 (−2000 to +500 bp relative to the TSS). *N* denotes successfully queried ChIP-seq datasets, not independent patients; *n* denotes datasets containing a promoter peak. Frequencies are descriptive; percentages are omitted when *N* < 5. The primary motif–ChIP analysis was restricted to strict-tier JASPAR motifs (*p* < 1 × 10^−4^). A motif was considered ChIP-overlapping when an AR-promoter ChIP-seq peak for the same TF intersected the corresponding motif interval. Supportive-tier motifs (1 × 10^−4^ ≤ *p* < 5 × 10^−4^) are shown separately as descriptive spatial overlaps and are not classified as primary strict-tier motif–ChIP overlaps. For FOXA1, the −751, −717, and −685 bp motifs form a compact cluster that is typically intersected by the same broad ChIP-seq peak; therefore, site-specific counts are not additive. Two E2F1 datasets and eight ERG datasets returned query errors and were excluded from the denominators. For E2F1/PC-3, neither retrieved dataset was successfully queried. The ChIP-Atlas metadata labels “PrEC” and “Primary prostate epithelial cells” were combined as “Primary prostate epithelial cells (PrEC)” for presentation. TFAP2C had no eligible ChIP-seq datasets in the supplied analysis. —, no selected JASPAR motif with same-TF ChIP-seq overlap. Abbreviations: ChIP, chromatin immunoprecipitation; ChIP-seq, ChIP followed by sequencing; mCRPC, metastatic castration-resistant prostate cancer; PrEC, primary prostate epithelial cells; TF, transcription factor; TSS, transcription start site; bp, base pairs. JASPAR is the TF-binding profile database.

**Table 6:** ChIP-seq peak regions within the AR promoter not overlapping the selected JASPAR motifs in prostate-derived cell models and mCRPC metastatic tissues (ChIP-Atlas *q* < 1 × 10^−5^).

TF	Biological Model/Tissue	Merged ChIP-Seq Peak Region (hg38)	Position Relative to TSS	Supporting Datasets, *n*/*N* (%)	Supporting SRX Datasets
FOXA1	VCaP	chrX: 67,543,678–67,544,296	−343 to +275 bp	2/49 (4.1%)	SRX17794370, SRX17794372
FOXA1	VCaP	chrX: 67,544,430–67,544,676	+409 to +655 bp	1/49 (2.0%)	SRX17794370
FOXA1	mCRPC metastasis—lung	chrX: 67,543,676–67,544,115	−345 to +94 bp	1/1	SRX8453148
HOXB13	VCaP	chrX: 67,543,950–67,544,196	−71 to +175 bp	1/12 (8.3%)	SRX24015774
AR	VCaP	chrX: 67,543,200–67,543,390	−821 to −631 bp	1/123 (0.8%)	SRX173202
AR	VCaP	chrX: 67,543,713–67,544,287	−308 to +266 bp	19/123 (15.4%)	SRX099182, SRX1569768, SRX1629233, SRX17042313, SRX17042314, SRX17042315, SRX17042316, SRX173202, SRX471848, SRX471850, SRX471855, SRX471856, SRX595677, SRX8089614, SRX8089615, SRX8089618, SRX8089619, SRX8089620, SRX8089621
AR	VCaP	chrX: 67,544,471–67,544,695	+450 to +674 bp	4/123 (3.3%)	SRX17042313, SRX17042314, SRX17042315, SRX17042316
AR	LNCaP	chrX: 67,543,201–67,543,418	−820 to −603 bp	4/323 (1.2%)	SRX21141305, SRX21141306, SRX9468652, SRX9468663
AR	LNCaP	chrX: 67,543,828–67,544,682	−193 to +661 bp	4/323 (1.2%)	SRX5286305, SRX5286307, SRX5286309, SRX5577161
AR	mCRPC metastasis—liver	chrX: 67,543,192–67,543,419	−829 to −602 bp	1/1	SRX8453143
AR	mCRPC metastasis—liver	chrX: 67,543,756–67,544,273	−265 to +252 bp	1/1	SRX8453143
AR	mCRPC metastasis—lymph node	chrX: 67,543,206–67,543,496	−815 to −525 bp	1/2	SRX8453140
AR	mCRPC metastasis—lymph node	chrX: 67,543,721–67,544,325	−300 to +304 bp	2/2	SRX8453140, SRX8453142
AR	mCRPC metastasis—lung	chrX: 67,543,763–67,544,191	−258 to +170 bp	1/1	SRX8453141
NR3C1/GR	VCaP	chrX: 67,543,713–67,544,241	−308 to +220 bp	1/26 (3.8%)	SRX173204
E2F1	LNCaP	chrX: 67,543,786–67,544,225	−235 to +204 bp	2/11 (18.2%)	SRX2565135, SRX8706937

Notes: Only successfully queried datasets with an AR-promoter ChIP-seq peak and no overlap with any selected JASPAR motif for the same TF are included. Peak coordinates are reported in hg38. The AR TSS used for relative coordinates is chrX:67,544,021; the analyzed promoter window was −2000 to +500 bp relative to the TSS. ChIP-seq defines an enriched peak interval rather than a nucleotide-resolution binding site. Overlapping peak calls from datasets of the same TF and biological model were merged into a single region; *n*/*N* is the number of datasets supporting that merged region divided by all successfully queried datasets for the same TF/model. Because the original ChIP-Atlas peak interval is reported in full when it intersects the query window, a called peak may extend beyond the nominal −2000 to +500 bp promoter window. Selected JASPAR motifs used for exclusion: FOXA1—MA0148.5: −1006, −993, −751, −717, −685 bp; HOXB13—MA0901.3: −1939, −1829, −1500, −698 bp; AR—MA0007.2: −1768, −1694, −1447, −1091 bp; NR3C1/GR—MA0113.2: −1738, −1695, −1448, −1090, −564 bp; E2F1—MA0024.3: −727, −264 bp. Abbreviations: ChIP-seq, chromatin immunoprecipitation followed by sequencing; mCRPC, metastatic castration-resistant prostate cancer; TF, transcription factor; TSS, transcription start site; bp, base pairs. JASPAR is the TF-binding profile database.

**Table 7:** GSE211138 VCaP AR/Input and IgG/Input datasets used for quantitative assessment of the proximal AR signal.

Assay	Condition	Rep.	SRX	GSM	Matched Input GSM	Role in Comparison
AR	DMSO + DHT	1	SRX17042313	GSM6451160	GSM6451172	Included in the four-dataset AR/Input range; directly treatment-matched to the DMSO+DHT IgG benchmark.
AR	DMSO + DHT	2	SRX17042314	GSM6451161	GSM6451172	Included in the four-dataset AR/Input range; directly treatment-matched to the DMSO+DHT IgG benchmark.
AR	ML792 + DHT	1	SRX17042315	GSM6451162	GSM6451173	Included in the four-dataset AR/Input range; not directly condition-matched to the IgG benchmark.
AR	ML792 + DHT	2	SRX17042316	GSM6451163	GSM6451174	Included in the four-dataset AR/Input range; not directly condition-matched to the IgG benchmark.
IgG	DMSO + DHT	1	—	GSM7031347	GSM7031351	Benchmark for nonspecific immunoprecipitation under the DMSO+DHT condition.

Abbreviations: DHT, dihydrotestosterone; DMSO, dimethyl sulfoxide; IgG, immunoglobulin G; Rep., replicate; GSM, Gene Expression Omnibus sample accession; SRX, Sequence Read Archive experiment accession. Input denotes the matched non-immunoprecipitated control; ML792 is the treatment compound specified in the source dataset.

**Table 8:** Peak-call screening of H3K27ac within the AR promoter across prostate-derived models and clinical tissues (AR promoter: hg38 chrX:67,542,021–67,544,521; −2000 to +500 bp relative to the TSS; ChIP-Atlas *q* < 1 × 10^−5^).

Biological Model/Tissue	H3K27ac Promoter Peak, *n*/*N* (%)	H3K27ac Proximal Overlap *, *n*/*N* Positive (%)
All datasets	145/516 (28.1%)	137/145 (94.5%)
VCaP	27/27 (100.0%)	26/27 (96.3%)
C4-2B	4/4	4/4
C4-2B-ER	2/2	2/2
C4-2	6/14 (42.9%)	6/6 (100.0%)
LNCaP	56/146 (38.4%)	50/56 (89.3%)
22Rv1	9/62 (14.5%)	9/9 (100.0%)
PC-3	1/37 (2.7%)	1/1
DU145	0/8 (0.0%)	—
RWPE-1	0/8 (0.0%)	—
RWPE-2	0/2	—
PrEC	0/2	—
Primary prostate epithelial cells	0/2	—
Primary prostate gland (ENCODE)	1/6 (16.7%)	1/1
Primary prostate tissue (normal, *N*)	2/38 (5.3%)	2/2
Primary prostate tissue (tumour, T)	35/152 (23.0%)	34/35 (97.1%)
mCRPC metastasis: liver	2/3	2/2
mCRPC metastasis: hilar lymph node	0/1	—
mCRPC metastasis: lung	0/1	—
mCRPC metastasis: perigastric lymph node	0/1	—

Notes: *n*/*N*, number of datasets containing at least one ChIP-seq peak overlapping the AR promoter divided by the total number of datasets for the corresponding biosample. Proximal H3K27ac overlap denotes H3K27ac-positive datasets containing a peak overlapping −280 to +100 bp relative to the AR TSS; the denominator is the number of H3K27ac-positive datasets for that biosample. Across all H3K27ac-positive datasets, 137/145 (94.5%) overlapped this proximal interval. The em dash (—) indicates that no H3K27ac-positive datasets were available for calculation of proximal overlap. C4-2B-ER, enzalutamide-resistant derivative of C4-2B; mCRPC, metastatic castration-resistant prostate cancer; TSS, transcription start site. Abbreviations: ChIP-seq, chromatin immunoprecipitation followed by sequencing; H3K27ac, acetylation of histone H3 at lysine 27; ENCODE, Encyclopedia of DNA Elements; *N*, normal tissue; T, tumor tissue.

**Table 9:** Study-first summary of Input-corrected H3K27me3 signal (ΔRPM) across predefined AR promoter/TSS windows.

Biological Model/Reference	*n*	Study Blocks	Promoter −2/+0.5 kb	TSS ±2 kb	TSS ±5 kb	TSS ±10 kb
Primary normal prostate tissue	4	1	−0.0727	−0.0476	−0.0099	−0.0091
PrEC (primary cultured epithelial cells)	1	1	0.0108	0.0178	0.0162	0.0096
RWPE-1 (GSE63094; immortalized benign)	2 *	1	0.0600	0.1400	0.0750	0.0400
Primary PCa GS6	2	1	−0.0289	−0.0256	−0.0049	−0.0070
Primary PCa GS8	4	1	−0.0202	−0.0178	−0.0121	−0.0044
LNCaP AR-dependent	5	4	−0.0083	−0.0082	−0.0070	−0.0060
LNCaP AI/CRPC	5	3	−0.0151	−0.0185	−0.0124	−0.0171
DU145	3	2	−0.0030	−0.0023	−0.0016	−0.0023
PC-3	3	3	0.0545	0.0670	0.0492	0.0277
mCRPC metastases	4	1	−0.0295	−0.0249	−0.0222	−0.0344

Values are ΔRPM = RPM_ChIP_ − RPM_Input_. For the 31 matched pairs, sample profiles were first summarized within each study block and then across study blocks to reduce weighting by studies with more replicates. Positive values indicate ChIP signal above matched Input; negative values indicate higher normalized Input signal in the specified window. * RWPE-1 comprises two H3K27me3 ChIP replicates sharing one matched Input and was analyzed separately from the 31-pair study-first dataset. Values are descriptive and should not be interpreted as spike-in-calibrated absolute differences between independent studies. Abbreviations: AI, androgen-independent; ChIP, chromatin immunoprecipitation; CRPC, castration-resistant prostate cancer; GS, Gleason score; H3K27me3, trimethylation of histone H3 at lysine 27; mCRPC, metastatic CRPC; PCa, prostate cancer; PrEC, primary prostate epithelial cells; RPM, reads per million; TSS, transcription start site; kb, kilobases. *n* denotes matched ChIP/Input pairs, except for RWPE-1, where it denotes the two ChIP replicates sharing one Input.

**Table 10:** Sequencing-depth-normalized enrichment of ncPRC1.1-associated components and H2AK119ub1 in distal and proximal regions of the AR promoter in PC-3 cells.

ChIP-seq Target	FOXA1 Motif Cluster (−751 to −678 bp)	Proximal Promoter (−280 to +100 bp)
KDM2B	0.825	4.952
PCGF1	2.931	3.957
RNF2	1.475	2.655
H2AK119ub1, replicate 1	2.329	8.852
H2AK119ub1, replicate 2	0.770	8.773

Values represent regional ChIP enrichment normalized to sequencing depth and the shared Input control (GSM3820351). Uniquely aligned single-end reads were directionally extended to 120 bp. Abbreviations: ChIP, chromatin immunoprecipitation; ChIP-seq, ChIP followed by sequencing; H2AK119ub1, monoubiquitination of histone H2A at lysine 119; ncPRC1.1, non-canonical Polycomb repressive complex 1.1; bp, base pairs.

## Data Availability

No new primary datasets were generated in this study. All source data analyzed are publicly available from the repositories and sources cited in the Materials and Methods section, including TCGA-PRAD, Gene Expression Omnibus (GEO; GSE158598, GSE130408, and GSE131829), ChIP-Atlas, JASPAR, and the WCDT mCRPC dataset described in the cited source. The analysis code, processed numerical inputs, dataset manifests, and supporting tables used to reproduce and verify the principal computational analyses are publicly available in Zenodo at https://doi.org/10.5281/zenodo.22957702 (accessed on 29 September 2026).

## Supplementary Materials

The following supporting information can be downloaded at: https://www.mdpi.com/article/10.3390/ijms27198822/s1.
