# Supplementary material for: Spatial and Epigenetic Polarity of the AR Promoter During Prostate Cancer Progression: An In Silico Analysis
Source: Int J Mol Sci. 2026 Oct 2;27(19):8822. doi: 10.3390/ijms27198822 (PMC13665174; doi:10.3390/ijms27198822)
Supplement: Supplementary file 1 [file ijms-27-08822-s001.zip › ijms-4583863-supplementary.pdf]

Supplementary Materials

Spatial and Epigenetic Polarity of the AR Promoter During Prostate Cancer Progression: An In Silico Analysis

Aleksey M. Krasnyi, Roman A. Ivanov, and Ekaterina Minskaia

Translational Medicine Research Center, Sirius University of Science and Technology, Federal Territory Sirius, Russia
Correspondence: alexred@list.ru

This supplement provides methylation–expression associations derived from matched samples, sequence-composition controls for motif analysis, and the complete catalog of predicted AR promoter motif clusters (Tables S1–S7).

S1. AR promoter methylation and AR expression

Table S1. Association between AR promoter CpG methylation and AR mRNA expression in TCGA-PRAD primary tumors.

CpG	Position relative to TSS, bp	n	Spearman ρ	95% bootstrap CI	p	BH q	Linear R², %
cg26003280	−1396	497	0.136	0.049 to 0.222	0.0023	0.0174	0.60
cg25686125	−854	496	0.006	−0.076 to 0.095	0.8854	0.9706	0.68
cg26014066	−769	497	−0.089	−0.175 to −0.000	0.0465	0.1395	1.20
cg11189761	−750	497	−0.018	−0.107 to 0.069	0.6845	0.8556	0.34
cg04755662	−504	497	−0.005	−0.088 to 0.077	0.9151	0.9706	0.23
cg14807303	−418	497	0.002	−0.085 to 0.090	0.9706	0.9706	0.32
cg12156154	−416	497	0.025	−0.062 to 0.114	0.5805	0.7916	0.07
cg12294334	−362	497	0.119	0.023 to 0.210	0.0077	0.0288	0.03
cg10164191	−342	497	0.038	−0.046 to 0.121	0.3925	0.6542	0.09
cg12180028	−259	497	−0.041	−0.128 to 0.047	0.3591	0.6542	0.50
cg20878850	−178	497	−0.042	−0.130 to 0.044	0.3514	0.6542	0.24
cg25085925	−172	497	−0.131	−0.218 to −0.042	0.0036	0.0178	0.34
cg08063601	+79	497	0.041	−0.046 to 0.130	0.3623	0.6542	0.10
cg16679837	+137	497	−0.190	−0.275 to −0.103	2.01×10^{-5}	0.0003	2.10
cg05786601	+263	497	0.025	−0.065 to 0.116	0.5804	0.7916	<0.01

Note. Spearman correlations were calculated between CpG β-values and AR RNA expression in sample-matched TCGA-PRAD primary tumors. P values were adjusted across the 15 prespecified CpGs using the Benjamini–Hochberg procedure. Confidence intervals for Spearman ρ were obtained from 2,000 bootstrap resamples. Linear R^2 is the coefficient of determination from a separate single-predictor linear regression and is not the square of Spearman ρ. For the joint linear model containing all 15 CpGs (n = 496), training R^2 = 0.0838, adjusted R^2 = 0.0551, and mean R^2 across 20 repeats of five-fold cross-validation = −0.00114. Positions are relative to the AR TSS at chrX:67,544,021 (GRCh38/hg38); genomic positions use a 1-based coordinate system. n, number of matched tumor samples; CI, confidence interval; BH, Benjamini–Hochberg; q, BH-adjusted p-value.

S2. Supplementary Methods

S2.1. Sequence composition controls for JASPAR motif analysis

Sequence-composition controls were performed as sensitivity analyses to evaluate whether the abundance of JASPAR motif matches within the AR promoter was greater than expected from sequence composition alone. These controls were not used to infer transcription-factor occupancy; the JASPAR scan remained a sequence-level mapping step for integration with ChIP-seq and epigenomic data.

The observed sequence was the 2501-bp AR promoter interval from −2000 to +500 bp relative to the transcription start site (TSS). The exact scanner used for the main analysis was retained: 24 transcription factors represented by 31 JASPAR profiles, promoter-specific mononucleotide background frequencies, a pseudocount of 0.8, PSSM score distributions calculated with 10,000 discrete levels, a strict threshold of $p < 1 \times 10^{-4}$, and an overall inclusion threshold of $p < 5 \times 10^{-4}$. Overlapping matches were clustered using the same rules as in the primary analysis. Re-analysis of the observed sequence reproduced exactly 104 clusters (38 strict and 66 supportive).

S2.2. Dinucleotide preserving permutation control

As a synthetic-sequence sensitivity control, 10,000 randomized sequences were generated by an Euler-trail procedure that preserved the exact counts of all 16 dinucleotides across the 2501-bp promoter. Each permuted sequence was scanned with the same JASPAR scoring and clustering procedure as the observed promoter. Empirical one-sided enrichment p-values were calculated as (1 + number of null sequences with a cluster count greater than or equal to the observed count)/(N + 1). Benjamini–Hochberg (BH) correction was applied across the 24 TF-level tests and, separately, across the 31 JASPAR matrix-level tests.

S2.3. Segment specific GC matched genomic control

Because the AR promoter is compositionally heterogeneous, the genomic control preserved its distal-to-proximal GC architecture. Real human TSS-centered promoter sequences (−2000 to +500 bp; GRCh38) were obtained from EPDnew and divided identically to the AR promoter into distal (−2000 to −281 bp) and proximal (−280 to +500 bp) segments. Before motif results were examined, the matching rule was fixed at ±3.0 percentage points of the AR GC content in both segments simultaneously. This yielded 1,083 matched promoters. CpG density and CpG observed/expected were calculated as diagnostics but were not used as matching criteria.

All matched promoters were scanned with the same 31 JASPAR profiles and the same fixed scoring procedure used for the observed AR sequence. Empirical enrichment was evaluated for the complete promoter and separately for distal and proximal segments. BH correction was applied within each region across 24 TF-level tests and, separately, across 31 matrix-level tests.

S3. Supplementary Results

S3.1. Sequence composition sensitivity analyses

The total number of AR promoter motif clusters was not increased relative to either control at the global level. In the dinucleotide-preserving control, 104 observed clusters were compared with a null mean of 94.3 (95% null interval, 71–119; empirical p = 0.219). In the GC-matched genomic control, 104 observed clusters were compared with a mean of 110.8 among 1,083 matched human promoters (95% interval, 84–140; empirical p = 0.688). The distal and proximal AR segments likewise did not show significant global enrichment relative to the matched-promoter background.

The dinucleotide-preserving control identified SP1 and KLF4 as significant after BH correction at the TF and matrix levels. These signals were not reproduced with the GC-matched real-promoter control: no TF and no individual JASPAR matrix remained significant after BH correction for the whole promoter, distal segment, or proximal segment. In the proximal segment, ERG and KLF4 showed nominal enrichment (TF-level p = 0.012 and 0.020, respectively), but neither survived BH correction (q = 0.244 for both; matrix-level q = 0.315 for each). This difference between synthetic and genomic controls indicates that apparent motif over-representation is sensitive to the choice of sequence background.

Accordingly, motif abundance was not used as evidence of regulatory occupancy. JASPAR matches were retained as candidate sequence positions for spatial integration with ChIP-seq and epigenomic data. Zero observed matches for MYC, NFKB1, HIF1A, and HIF2A were treated descriptively and were not interpreted as evidence of biological absence or depletion.

Table S2. Global motif-cluster abundance relative to the two sequence-composition controls.

Control	Region	Observed	Background N	Mean	Median	95% interval	Fold	Empirical p
GC-matched real promoters	Whole	104	1083	110.77	110.00	84–140	0.94	0.688
GC-matched real promoters	Distal	53	1083	62.84	63.00	43–85	0.84	0.834
GC-matched real promoters	Proximal	51	1083	47.93	47.00	31–68	1.06	0.369
Dinucleotide-preserving permutations	Whole	104	10000	94.32	94.00	71–119	1.10	0.219

Empirical p-values are one-sided tests of over-representation. Observed, number of AR motif clusters; background N, number of control sequences; mean, median and 95% interval summarize background cluster counts; fold, observed count divided by the background mean. Whole, −2000 to +500 bp; distal, −2000 to −281 bp; proximal, −280 to +500 bp relative to the AR TSS.

Table S3. Whole-promoter TF-level enrichment under GC-matched and dinucleotide-preserving controls.

TF	AR clusters	GC mean	GC fold	GC p	GC BH q	Dinuc mean	Dinuc fold	Dinuc p	Dinuc BH q
SP1	13	10.39	1.25	0.239	0.861	4.92	2.64	0.0005	**0.0060**
FOXA1	5	3.50	1.43	0.282	0.861	1.87	2.67	0.039	0.236
GATA2	2	2.03	0.99	0.594	1.000	1.87	1.07	0.556	1.000
HOXB13	4	5.68	0.70	0.813	1.000	3.16	1.27	0.386	1.000
TFAP2C	10	14.63	0.68	0.859	1.000	12.33	0.81	0.743	1.000
NKX3-1	1	2.25	0.44	0.888	1.000	2.32	0.43	0.905	1.000
AR	4	3.27	1.22	0.408	0.979	3.30	1.21	0.420	1.000
NR3C1	6	3.03	1.98	0.107	0.861	3.17	1.89	0.111	0.535
CTCF	4	9.87	0.41	0.992	1.000	9.85	0.41	0.991	1.000
E2F1	4	1.63	2.46	0.156	0.861	1.75	2.28	0.179	0.615
MYC	0	2.22	0.00	1.000	1.000	1.33	0.00	1.000	1.000
KLF4	11	6.59	1.67	0.082	0.861	3.08	3.57	1.00e-04	**0.0024**
KLF5	9	6.73	1.34	0.253	0.861	3.71	2.43	0.0092	0.074
ERG	8	5.42	1.48	0.184	0.861	5.15	1.55	0.144	0.575
ETS1	4	2.69	1.48	0.287	0.861	2.76	1.45	0.296	0.889
JUN	1	5.33	0.19	0.985	1.000	4.37	0.23	0.978	1.000
STAT1	4	4.01	1.00	0.549	1.000	4.73	0.85	0.670	1.000
STAT5	9	7.50	1.20	0.364	0.972	9.29	0.97	0.563	1.000
RELA	3	2.87	1.04	0.529	1.000	3.88	0.77	0.723	1.000
NFKB1	0	3.66	0.00	1.000	1.000	4.18	0.00	1.000	1.000
NFKB2	1	3.29	0.30	0.856	1.000	3.85	0.26	0.902	1.000
HIF1A	0	1.03	0.00	1.000	1.000	0.57	0.00	1.000	1.000
HIF2A	0	1.27	0.00	1.000	1.000	0.55	0.00	1.000	1.000
TP53	1	1.88	0.53	0.713	1.000	2.30	0.43	0.764	1.000

Note. GC = segment-specific GC-matched real-promoter background (N = 1,083); Dinuc = 10,000 exact dinucleotide-preserving permutations. Bold q-values indicate FDR < 0.05. TF, transcription factor; BH, Benjamini–Hochberg; mean, background mean cluster count; fold, observed/background mean. Empirical p-values are one-sided; q-values are adjusted across the 24 TFs separately for each control.

Table S4. Whole-promoter matrix-level enrichment with BH correction across 31 JASPAR profiles.

TF	JASPAR profile	AR clusters	GC p	GC BH q	Dinuc p	Dinuc BH q
SP1	MA0079.5	13	0.239	0.889	0.0005	**0.0077**
FOXA1	MA0148.5	5	0.282	0.889	0.039	0.305
GATA2	MA0036.4	2	0.594	1.000	0.556	1.000
HOXB13	MA0901.3	4	0.813	1.000	0.386	1.000
TFAP2C	MA0524.3	4	0.752	1.000	0.735	1.000
TFAP2C	MA0814.3	5	0.827	1.000	0.544	1.000
TFAP2C	MA0815.1	2	0.927	1.000	0.914	1.000
NKX3-1	MA0124.3	1	0.888	1.000	0.905	1.000
AR	MA0007.2	4	0.408	1.000	0.420	1.000
NR3C1	MA0113.2	6	0.107	0.889	0.111	0.691
CTCF	MA0139.2	1	0.989	1.000	0.988	1.000
CTCF	MA1929.2	2	0.932	1.000	0.927	1.000
CTCF	MA1930.2	2	0.918	1.000	0.913	1.000
E2F1	MA0024.3	4	0.156	0.889	0.179	0.794
MYC	MA0147.4	0	1.000	1.000	1.000	1.000
KLF4	MA0039.5	11	0.082	0.889	1.00e-04	**0.0031**
KLF5	MA0599.1	9	0.253	0.889	0.0092	0.095
ERG	MA0474.4	8	0.184	0.889	0.144	0.742
ETS1	MA0098.4	4	0.287	0.889	0.296	1.000
JUN	MA0488.2	0	1.000	1.000	1.000	1.000
JUN	MA0489.3	1	0.833	1.000	0.834	1.000
STAT1	MA0137.4	4	0.549	1.000	0.670	1.000
STAT5	MA0519.2	6	0.276	0.889	0.393	1.000
STAT5	MA1624.2	7	0.126	0.889	0.266	1.000
STAT5	MA1625.2	3	0.666	1.000	0.807	1.000
RELA	MA0107.1	3	0.529	1.000	0.723	1.000
NFKB1	MA0105.4	0	1.000	1.000	1.000	1.000
NFKB2	MA0778.2	1	0.856	1.000	0.902	1.000
HIF1A	MA1106.2	0	1.000	1.000	1.000	1.000
HIF2A	MA2325.1	0	1.000	1.000	1.000	1.000
TP53	MA0106.3	1	0.713	1.000	0.764	1.000

Note. No JASPAR matrix was significant after BH correction in the GC-matched real-promoter control. SP1/MA0079.5 and KLF4/MA0039.5 were significant only in the dinucleotide-preserving control. GC, GC-matched real-promoter control (N = 1,083); Dinuc, dinucleotide-preserving permutations (N = 10,000); BH, Benjamini–Hochberg. Bold q-values indicate FDR < 0.05.

Table S5. Composition diagnostics for the segment-specific GC-matched promoter background.

Region	Metric	AR target	N	Background mean	Median	95% interval
Distal	GC (%)	42.79	1083	43.15	43.31	40.06–45.70
Distal	CpG/kb	9.88	1083	17.48	16.86	6.40–32.56
Distal	CpG O/E	0.216	1083	0.376	0.369	0.146–0.663
Proximal	GC (%)	65.56	1083	65.54	65.43	62.74–68.37
Proximal	CpG/kb	62.74	1083	84.08	84.51	56.34–110.12
Proximal	CpG O/E	0.588	1083	0.790	0.794	0.543–0.996

Note. CpG characteristics were reported diagnostically and were not used for background selection. O/E, observed/expected. N, number of matched promoters; mean, median and 95% interval summarize the matched-promoter background. Distal, −2000 to −281 bp; proximal, −280 to +500 bp relative to the AR TSS.

Table S6. Nominal proximal-segment signals in the GC-matched control.

TF	Profile	AR clusters	Background mean	Fold	TF p	TF BH q	Matrix BH q
ERG	MA0474.4	6	1.92	3.12	0.012	0.244	0.315
KLF4	MA0039.5	10	4.23	2.37	0.020	0.244	0.315

Note. These nominal p-values did not remain significant after BH correction and were therefore not interpreted as motif enrichment. Background mean, mean cluster count among 1,083 matched promoters; fold, observed/background mean; TF, transcription factor; BH, Benjamini–Hochberg. TF-level and matrix-level q-values were adjusted across 24 TFs and 31 JASPAR profiles, respectively.

S4. Predicted AR promoter motif clusters

Table S7. Predicted transcription-factor motif clusters in the AR promoter.

All 104 retained clusters are listed (38 strict and 66 supportive). Genomic coordinates refer to chromosome X, GRCh38/hg38, and are 1-based inclusive. Relative coordinates are calculated from the AR TSS at chrX:67,544,021. Cluster boundaries span merged overlapping predictions for the same TF and strand. The representative site is the match with the lowest nominal p-value, with the highest PSSM score used to resolve ties. Strict: $p < 10^{-4}$; supportive: $10^{-4} \leq p < 5 \times 10^{-4}$. The p-value describes the representative sequence match and does not measure cluster enrichment or experimental binding support.

ID	TF	JASPAR matrix	Strand	Cluster hg38 start–end	Cluster relative start–end (bp)	Site hg38 start–end	Site relative start–end (bp)	Nominal p	Tier	Representative sequence
M001	SP1	MA0079.5	-	67543101–67543109	-920 to -912	67543101–67543109	-920 to -912	4.552223E-04	supportive	GTGGAGGAG
M002	SP1	MA0079.5	+	67543457–67543465	-564 to -556	67543457–67543465	-564 to -556	3.213309E-04	supportive	GGGGCTGAG
M003	SP1	MA0079.5	-	67543833–67543848	-188 to -173	67543833–67543841	-188 to -180	4.314512E-04	supportive	GGGCAGGAG
M004	SP1	MA0079.5	+	67543900–67543918	-121 to -103	67543905–67543913	-116 to -108	5.707229E-06	strict	GGGGAGGGG
M005	SP1	MA0079.5	+	67543925–67543938	-96 to -83	67543925–67543933	-96 to -88	5.707229E-06	strict	GGGGAGGGG
M006	SP1	MA0079.5	+	67543974–67543982	-47 to -39	67543974–67543982	-47 to -39	1.161662E-05	strict	GGGGCGGGA
M007	SP1	MA0079.5	-	67544013–67544028	-8 to 7	67544020–67544028	-1 to 7	8.479977E-05	strict	GGGGCGCTG
M008	SP1	MA0079.5	+	67544041–67544049	20 to 28	67544041–67544049	20 to 28	3.304499E-04	supportive	GGGGAGCCA
M009	SP1	MA0079.5	-	67544332–67544356	311 to 335	67544348–67544356	327 to 335	4.182344E-05	strict	GGGGAGGCG
M010	SP1	MA0079.5	-	67544367–67544380	346 to 359	67544372–67544380	351 to 359	5.707229E-06	strict	GGGGAGGGG
M011	SP1	MA0079.5	-	67544427–67544435	406 to 414	67544427–67544435	406 to 414	2.959881E-04	supportive	GGTGAGGGG
M012	SP1	MA0079.5	-	67544439–67544447	418 to 426	67544439–67544447	418 to 426	4.056387E-04	supportive	GGGGAGAAG
M013	SP1	MA0079.5	-	67544448–67544464	427 to 443	67544456–67544464	435 to 443	2.944930E-06	strict	GGGGCGGGG
M014	FOXA1	MA0148.5	-	67543015–67543022	-1006 to -999	67543015–67543022	-1006 to -999	1.285721E-04	supportive	GAAAACAT

ID	TF	JASPAR matrix	Strand	Cluster hg38 start–end	Cluster relative start–end (bp)	Site hg38 start–end	Site relative start–end (bp)	Nominal p	Tier	Representative sequence
M015	FOXA1	MA0148.5	-	67543028–67543041	-993 to -980	67543028–67543035	-993 to -986	4.007125E-04	supportive	GTAAACCT
M016	FOXA1	MA0148.5	-	67543270–67543277	-751 to -744	67543270–67543277	-751 to -744	4.283038E-04	supportive	GTAAACGT
M017	FOXA1	MA0148.5	-	67543304–67543311	-717 to -710	67543304–67543311	-717 to -710	1.285721E-04	supportive	GTACACAT
M018	FOXA1	MA0148.5	-	67543336–67543343	-685 to -678	67543336–67543343	-685 to -678	5.529944E-05	strict	GTAAACAC
M019	GATA2	MA0036.4	-	67542837–67542843	-1184 to -1178	67542837–67542843	-1184 to -1178	1.402023E-04	supportive	TTTATCT
M020	GATA2	MA0036.4	+	67543609–67543615	-412 to -406	67543609–67543615	-412 to -406	7.250684E-05	strict	CTTATCA
M021	HOXB13	MA0901.3	-	67542082–67542090	-1939 to -1931	67542082–67542090	-1939 to -1931	7.346059E-05	strict	CCAATATAA
M022	HOXB13	MA0901.3	-	67542192–67542200	-1829 to -1821	67542192–67542200	-1829 to -1821	3.739891E-04	supportive	TGAATAAAA
M023	HOXB13	MA0901.3	+	67542520–67542529	-1501 to -1492	67542521–67542529	-1500 to -1492	3.487410E-05	strict	CCATTAAAA
M024	HOXB13	MA0901.3	-	67543323–67543331	-698 to -690	67543323–67543331	-698 to -690	3.346040E-04	supportive	ACACTAAAA
M025	TFAP2C	MA0814.3	-	67542552–67542560	-1469 to -1461	67542552–67542560	-1469 to -1461	1.820257E-04	supportive	ACCTGAGGA
M026	TFAP2C	MA0524.3	-	67542769–67542779	-1252 to -1242	67542769–67542779	-1252 to -1242	2.390025E-04	supportive	ACCTAAAGGCA
M027	TFAP2C	MA0814.3	+	67543459–67543467	-562 to -554	67543459–67543467	-562 to -554	1.854447E-04	supportive	GGCTGAGGG
M028	TFAP2C	MA0524.3	+	67543737–67543747	-284 to -274	67543737–67543747	-284 to -274	3.367788E-04	supportive	GCCTCCAGGAA
M029	TFAP2C	MA0815.1	+	67543965–67543978	-56 to -43	67543965–67543977	-56 to -44	2.166807E-04	supportive	GGCCCGGTGGGGG
M030	TFAP2C	MA0815.1	-	67543965–67543978	-56 to -43	67543965–67543977	-56 to -44	2.475124E-04	supportive	CCCCCACCGGGCC
M031	TFAP2C	MA0524.3	-	67544081–67544091	60 to 70	67544081–67544091	60 to 70	2.355442E-04	supportive	GCCTCTGGGCT
M032	TFAP2C	MA0524.3	+	67544082–67544092	61 to 71	67544082–67544092	61 to 71	1.959648E-05	strict	GCCCAGAGGCA
M033	TFAP2C	MA0814.3	-	67544170–67544178	149 to 157	67544170–67544178	149 to 157	4.678661E-04	supportive	GGCTGGGGC

ID	TF	JASPAR matrix	Strand	Cluster hg38 start–end	Cluster relative start–end (bp)	Site hg38 start–end	Site relative start–end (bp)	Nominal p	Tier	Representative sequence
M034	TFAP2C	MA0814.3	-	67544400–67544408	379 to 387	67544400–67544408	379 to 387	1.356490E-04	supportive	GACTGAGGC
M035	NKX3-1	MA0124.3	+	67542481–67542487	-1540 to -1534	67542481–67542487	-1540 to -1534	2.657508E-04	supportive	CTACTTA
M036	AR	MA0007.2	-	67542253–67542267	-1768 to -1754	67542253–67542267	-1768 to -1754	1.863947E-04	supportive	GAGGACACATAGACA
M037	AR	MA0007.2	-	67542327–67542341	-1694 to -1680	67542327–67542341	-1694 to -1680	1.207403E-04	supportive	GAGAACTATGTGTCT
M038	AR	MA0007.2	-	67542574–67542588	-1447 to -1433	67542574–67542588	-1447 to -1433	5.865550E-05	strict	CAGAACACAAAGGCC
M039	AR	MA0007.2	+	67542930–67542944	-1091 to -1077	67542930–67542944	-1091 to -1077	1.105627E-04	supportive	TGGAACAGAGAGCCT
M040	NR3C1	MA0113.2	-	67542283–67542297	-1738 to -1724	67542283–67542297	-1738 to -1724	2.580462E-04	supportive	ATCACTGAATGAACA
M041	NR3C1	MA0113.2	-	67542326–67542340	-1695 to -1681	67542326–67542340	-1695 to -1681	8.406125E-05	strict	AGAACTATGTGTCTA
M042	NR3C1	MA0113.2	-	67542573–67542587	-1448 to -1434	67542573–67542587	-1448 to -1434	1.033025E-04	supportive	AGAACACAAAGGCCT
M043	NR3C1	MA0113.2	+	67542931–67542945	-1090 to -1076	67542931–67542945	-1090 to -1076	2.049538E-04	supportive	GGAACAGAGAGCCTC
M044	NR3C1	MA0113.2	-	67542931–67542945	-1090 to -1076	67542931–67542945	-1090 to -1076	3.763877E-04	supportive	GAGGCTCTCTGTTCC
M045	NR3C1	MA0113.2	+	67543457–67543471	-564 to -550	67543457–67543471	-564 to -550	2.948619E-04	supportive	GGGGCTGAGGGTTCC
M046	CTCF	MA1929.2	-	67543154–67543184	-867 to -837	67543154–67543184	-867 to -837	4.161513E-04	supportive	ATATAGGGCTGCTTTTCGCCTCTTGATG GAC
M047	CTCF	MA0139.2	+	67543948–67543980	-73 to -41	67543966–67543980	-55 to -41	1.963321E-04	supportive	GCCCGGTGGGGGCGG
M048	CTCF	MA1929.2	-	67544022–67544053	1 to 32	67544023–67544053	2 to 32	2.294447E-04	supportive	AAGCTGGCTCCCCGGGATCTCGGAGGG GGCG
M049	CTCF	MA1930.2	+	67544130–67544162	109 to 141	67544130–67544162	109 to 141	4.885369E-04	supportive	CTCCAGTGCTGTACAGGAGCCGAAGGG ACGCAC
M050	E2F1	MA0024.3	+	67543294–67543305	-727 to -716	67543294–67543305	-727 to -716	1.561895E-04	supportive	TTGAGCGCCTAT
M051	E2F1	MA0024.3	-	67543294–67543305	-727 to -716	67543294–67543305	-727 to -716	5.999846E-05	strict	ATAGGCGCTCAA
M052	E2F1	MA0024.3	+	67543757–67543768	-264 to -253	67543757–67543768	-264 to -253	8.025585E-05	strict	CCTGGCGCCTAA

ID	TF	JASPAR matrix	Strand	Cluster hg38 start–end	Cluster relative start–end (bp)	Site hg38 start–end	Site relative start–end (bp)	Nominal p	Tier	Representative sequence
M053	E2F1	MA0024.3	-	67543757–67543768	-264 to -253	67543757–67543768	-264 to -253	3.162365E-05	strict	TTAGGCGCCAGG
M054	KLF4	MA0039.5	+	67543682–67543689	-339 to -332	67543682–67543689	-339 to -332	5.386720E-05	strict	CTCCACCC
M055	KLF4	MA0039.5	-	67543906–67543913	-115 to -108	67543906–67543913	-115 to -108	7.230797E-05	strict	CCCCTCCC
M056	KLF4	MA0039.5	-	67543926–67543933	-95 to -88	67543926–67543933	-95 to -88	7.230797E-05	strict	CCCCTCCC
M057	KLF4	MA0039.5	-	67543969–67543982	-52 to -39	67543969–67543976	-52 to -45	3.732182E-04	supportive	CCCCACCG
M058	KLF4	MA0039.5	+	67544171–67544178	150 to 157	67544171–67544178	150 to 157	2.675822E-04	supportive	CCCCAGCC
M059	KLF4	MA0039.5	+	67544340–67544352	319 to 331	67544340–67544347	319 to 326	5.386720E-05	strict	CTCCACCC
M060	KLF4	MA0039.5	+	67544355–67544362	334 to 341	67544355–67544362	334 to 341	1.835540E-05	strict	CCCCACCC
M061	KLF4	MA0039.5	+	67544372–67544379	351 to 358	67544372–67544379	351 to 358	7.230797E-05	strict	CCCCTCCC
M062	KLF4	MA0039.5	+	67544432–67544439	411 to 418	67544432–67544439	411 to 418	1.460666E-04	supportive	CACCACCC
M063	KLF4	MA0039.5	+	67544444–67544455	423 to 434	67544444–67544451	423 to 430	1.835540E-05	strict	CCCCACCC
M064	KLF4	MA0039.5	+	67544456–67544463	435 to 442	67544456–67544463	435 to 442	5.386720E-05	strict	CCCCGCCC
M065	KLF5	MA0599.1	+	67543665–67543690	-356 to -331	67543681–67543690	-340 to -331	1.739145E-04	supportive	CCTCCACCCT
M066	KLF5	MA0599.1	+	67543839–67543848	-182 to -173	67543839–67543848	-182 to -173	2.528905E-04	supportive	CCCTCGCCCA
M067	KLF5	MA0599.1	-	67543905–67543919	-116 to -102	67543905–67543914	-116 to -107	2.869768E-05	strict	TCCCCTCCCC
M068	KLF5	MA0599.1	-	67543925–67543939	-96 to -82	67543925–67543934	-96 to -87	2.869768E-05	strict	TCCCCTCCCC
M069	KLF5	MA0599.1	-	67543974–67543983	-47 to -38	67543974–67543983	-47 to -38	4.162974E-05	strict	GTCCCGCCCC
M070	KLF5	MA0599.1	+	67544331–67544353	310 to 332	67544339–67544348	318 to 327	1.182427E-04	supportive	TCTCCACCCC
M071	KLF5	MA0599.1	+	67544354–67544363	333 to 342	67544354–67544363	333 to 342	3.571956E-05	strict	CCCCCACCCT

ID	TF	JASPAR matrix	Strand	Cluster hg38 start–end	Cluster relative start–end (bp)	Site hg38 start–end	Site relative start–end (bp)	Nominal p	Tier	Representative sequence
M072	KLF5	MA0599.1	+	67544371–67544380	350 to 359	67544371–67544380	350 to 359	3.925467E-05	strict	CCCCCTCCCC
M073	KLF5	MA0599.1	+	67544455–67544464	434 to 443	67544455–67544464	434 to 443	2.025907E-05	strict	CCCCCGCCCC
M074	ERG	MA0474.4	+	67542532–67542541	-1489 to -1480	67542532–67542541	-1489 to -1480	9.784588E-05	strict	TCAGGAAATA
M075	ERG	MA0474.4	+	67543411–67543420	-610 to -601	67543411–67543420	-610 to -601	2.514180E-05	strict	ACAGGAAGAA
M076	ERG	MA0474.4	+	67543741–67543750	-280 to -271	67543741–67543750	-280 to -271	1.170868E-04	supportive	CCAGGAAATC
M077	ERG	MA0474.4	+	67543796–67543805	-225 to -216	67543796–67543805	-225 to -216	1.218992E-04	supportive	TCAGGAAGCA
M078	ERG	MA0474.4	+	67543917–67543926	-104 to -95	67543917–67543926	-104 to -95	2.352555E-04	supportive	AAAGGAAAGG
M079	ERG	MA0474.4	+	67543939–67543948	-82 to -73	67543939–67543948	-82 to -73	4.983361E-04	supportive	AAAGGAGGTG
M080	ERG	MA0474.4	-	67544243–67544252	222 to 231	67544243–67544252	222 to 231	1.664182E-04	supportive	AGAGGAAAGG
M081	ERG	MA0474.4	+	67544285–67544294	264 to 273	67544285–67544294	264 to 273	3.788197E-04	supportive	GGAGGAAGCA
M082	ETS1	MA0098.4	+	67542532–67542540	-1489 to -1481	67542532–67542540	-1489 to -1481	2.716153E-04	supportive	TCAGGAAAT
M083	ETS1	MA0098.4	+	67543411–67543419	-610 to -602	67543411–67543419	-610 to -602	3.063808E-04	supportive	ACAGGAAGA
M084	ETS1	MA0098.4	+	67543741–67543749	-280 to -272	67543741–67543749	-280 to -272	2.891929E-04	supportive	CCAGGAAAT
M085	ETS1	MA0098.4	+	67543796–67543804	-225 to -217	67543796–67543804	-225 to -217	3.931398E-04	supportive	TCAGGAAGC
M086	JUN	MA0489.3	-	67542631–67542638	-1390 to -1383	67542631–67542638	-1390 to -1383	3.519911E-04	supportive	AGACTCAT
M087	STAT1	MA0137.4	+	67542504–67542512	-1517 to -1509	67542504–67542512	-1517 to -1509	3.983510E-05	strict	TTCAAGGAA
M088	STAT1	MA0137.4	-	67542944–67542952	-1077 to -1069	67542944–67542952	-1077 to -1069	2.620353E-04	supportive	TTCTGGGGA
M089	STAT1	MA0137.4	-	67543468–67543476	-553 to -545	67543468–67543476	-553 to -545	1.058888E-04	supportive	CTCTAGGAA
M090	STAT1	MA0137.4	+	67543739–67543747	-282 to -274	67543739–67543747	-282 to -274	9.120435E-05	strict	CTCCAGGAA

ID	TF	JASPAR matrix	Strand	Cluster hg38 start–end	Cluster relative start–end (bp)	Site hg38 start–end	Site relative start–end (bp)	Nominal p	Tier	Representative sequence
M091	STAT5	MA1624.2	+	67542504–67542512	-1517 to -1509	67542504–67542512	-1517 to -1509	1.917530E-04	supportive	TTCAAGGAA
M092	STAT5	MA0519.2	-	67542504–67542512	-1517 to -1509	67542504–67542512	-1517 to -1509	1.089270E-04	supportive	TTCCTTGAA
M093	STAT5	MA0519.2	+	67542870–67542878	-1151 to -1143	67542870–67542878	-1151 to -1143	4.700170E-04	supportive	TTCTCTGTA
M094	STAT5	MA0519.2	+	67542944–67542952	-1077 to -1069	67542944–67542952	-1077 to -1069	5.360862E-05	strict	TCCCCAGAA
M095	STAT5	MA1624.2	-	67543452–67543460	-569 to -561	67543452–67543460	-569 to -561	4.029488E-04	supportive	CCCCAAGAA
M096	STAT5	MA0519.2	+	67543468–67543476	-553 to -545	67543468–67543476	-553 to -545	7.382242E-05	strict	TTCCTAGAG
M097	STAT5	MA1624.2	-	67543468–67543476	-553 to -545	67543468–67543476	-553 to -545	3.584990E-04	supportive	CTCTAGGAA
M098	STAT5	MA1624.2	+	67543739–67543747	-282 to -274	67543739–67543747	-282 to -274	5.153823E-05	strict	CTCCAGGAA
M099	STAT5	MA0519.2	-	67543739–67543747	-282 to -274	67543739–67543747	-282 to -274	1.558220E-04	supportive	TTCCTGGAG
M100	RELA	MA0107.1	-	67543400–67543409	-621 to -612	67543400–67543409	-621 to -612	1.589395E-04	supportive	TGGAATTGCC
M101	RELA	MA0107.1	-	67544296–67544305	275 to 284	67544296–67544305	275 to 284	2.005067E-04	supportive	GGCACTTTCC
M102	RELA	MA0107.1	-	67544501–67544510	480 to 489	67544501–67544510	480 to 489	1.020376E-04	supportive	GGGAGTTACC
M103	NFKB2	MA0778.2	+	67544500–67544510	479 to 489	67544500–67544510	479 to 489	4.958183E-04	supportive	AGGTAACTCCC
M104	TP53	MA0106.3	+	67543223–67543253	-798 to -768	67543223–67543240	-798 to -781	3.838264E-04	supportive	AATATGCGAAGGCAACTT

TF, transcription factor; TSS, transcription start site; PSSM, position-specific scoring matrix. Cluster and representative-site coordinates are reported separately. Nominal p-values are shown in scientific notation. Source: reproducibility archive, supporting_tables/motif_clusters_104.csv (https://doi.org/10.5281/zenodo.22957702). An Excel version of Table S7 is also supplied, including PSSM scores, supporting matrices, counts of merged predictions, CpG counts, and promoter-segment assignments.
